# Nonenergy Biomass Carbon Removal and Storage (BiCRS): Assessing Durability of Nongaseous Carbon Products Across Terrestrial Storage Fates

**DOI:** 10.1021/acs.chemrev.5c00618

**Published:** 2026-04-10

**Authors:** Sinéad M. Crotty, Peter W. Reiners, Leah K. Clayton, Edward Young, Andrew Jones, Melissa A. Cregger, Anne K. Starace, Anne E. Harman-Ware

**Affiliations:** † Carbon Containment Lab, 83-87 Audubon Street, New Haven, Connecticut 06510, United States; ‡ Biosciences Center, National Laboratory of the Rockies, Golden, Colorado 80401, United States; § Geosciences, University of Arizona, Tucson, Arizona 85721, United States; ∥ Charm Industrial, San Francisco California 94124, United States; ⊥ Carba, Inc., 7561 Corporate Way, Eden Prairie, Minnesota 55344, United States; # Biosciences Division, Oak Ridge National Laboratory, Oak Ridge, Tennessee 37830, United States,; ⊗ Catalytic Carbon Transformation and Scale Up Center, National Laboratory of the Rockies, Golden, Colorado 80401, United States

## Abstract

Biomass Carbon Removal and Storage, or BiCRS, pathways use plants or algae that remove carbon dioxide from the atmosphere through photosynthesis and store it underground or in long-lived products. While some BiCRS approaches generate an energy product, all BiCRS approaches generate a carbon product. A new subset of BiCRS approaches focus on the storage of these raw or converted carbon products for generation of carbon credits. However, the durability of these approaches is highly variable as carbon products vary widely in their “form” and the conditions of their “fate.” We organize our thinking about carbon products and their durability around these two primary axes. The durability of carbon product “forms” is mediated by chemical recalcitrance and ranges substantially across agricultural residues, municipal solid waste, woody biomass, and nongaseous products of thermochemical conversion (e.g., biochars and bio-oils). Meanwhile, terrestrial storage “fates” vary in the mechanism employed to stall decay, including surface storage, dry storage, shallow anoxic storage, and deep or geologic anoxic storage (or injection). Each mechanism has different implications for suitability with different feedstock forms as well as long-term risks. We present a framework for assessing durability of solid or liquid raw and conversion carbon products under terrestrial storage fates, highlighting knowns, unknowns, and research priorities moving forward.

## Graphical abstract

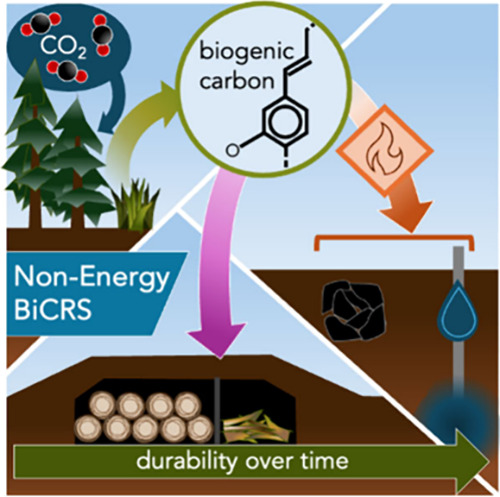

## 1 Introduction

Carbon dioxide removal (CDR) includes any human process that captures carbon dioxide (CO_2_) directly from the atmosphere and durably stores it in terrestrial, aquatic, or geologic systems, or in long-lived products.^1^ While this definition excludes natural removal processes lacking human intervention, it includes cases whereby human activities enhance natural processes.^2^ Conventional methods of CDR have been ongoing for the past several decades, and include processes such as afforestation and reforestation, peatland and coastal wetland restoration, and soil carbon sequestration. These conventional processes capture and store a combined total of approximately 2.2 billion metric tons of CO_2_ equivalents (MTCO_2_e) each year.^3,4^

Meanwhile, emerging technologies and novel methods including enhanced rock weathering, direct air carbon capture and storage, and bioenergy with carbon capture and storage (BECCS), are currently only achieving 1.3 million MTCO_2_e each year of CDR.^3^ Recent market analyses suggest a substantial increase in near-term demand for novel CDR, on the order of 36–180 million MTCO_2_e each year by 2030.^5^ This market demand far exceeds the currently available supply, and projections for future tons delivered.^3^

A core principle of high-quality CDR is that the storage of the atmospherically derived carbon must be durable—i.e., not quickly returned to the atmosphere on human time scales (i.e., decades).^3^ However, there is a wide variation as to what time frame constitutes durable storage in addition to the measurement, monitoring, reporting, and verification (MMRV) requirements sufficient to demonstrate this durability (see Appendix for criteria of high quality CDR). For example, contractual standards range from 10 to >100 years, while project developers are claiming durability from 1 year to 10 million years.^6^

The physical, chemical, and biological pathways of both storage and reversal vary widely depending on the nature of the storage reservoir (e.g., soil carbon, above and belowground biomass, geologic formation, ocean, or product-based), the form of stored carbon (e.g., organic biomass, biochar, inorganic carbonate, CO_2_), and the degree to which the system is open or closed to further carbon exchange. Open systems can be especially vulnerable to perturbation from phenomena including land use change or wildfire, microbial decomposition, or longer-term ocean circulation shifts—perturbations whose frequency and magnitude may also increase in response to warming, acidification, and other anthropogenic changes.^7–10^ In these systems, durability is not only a function of initial sequestration but also of the capacity to manage or buffer against re-emission of stored carbon, known as reversal, over time. For example, carbon stored as organic matter in biomass or soil may be lost through decay, combustion, or erosion within years to decades, unless active measures are taken to protect it.^11,12^

Given the vast variability across sectors and approaches for durability claims, carbon credit methodologies can require durability mechanisms (i.e., how the maintenance and protection of the stored carbon will be guaranteed for the set time period) and reversal management mechanisms (i.e., how risk of re-emission will be managed and remediated if realized).^6^ For these mechanisms to be successful, there needs to be a strong understanding of the expected timing and rate of re-emission, gas composition (e.g., CO_2_, CH_4_, etc.), and vulnerability to additional perturbation. Reversal management mechanisms in particular will be dependent on the storage reservoir.

One novel approach that is receiving increased attention for its potential to deliver durable and low-cost CDR is Biomass Carbon Removal and Storage, or BiCRS. BiCRS describes a series of pathways that use terrestrial plants or algae to remove CO_2_ from the atmosphere and store it underground or in long-lived products.^13^ BiCRS pathways are based on the “Aines Principle,” which theorizes that the value of using biomass for removing carbon from the atmosphere may exceed the energy value of that biomass.^13^ Recent technoeconomic assessments provide additional support for this theory.^14^

In the United States alone, recent estimates suggest that BiCRS can safely remove over 800 million MTCO_2_e annually by 2050 at a net cost under $100 per tonne.^15^ While many of the BiCRS approaches focus on developing technologies to valorize both energy and carbon storage (e.g., BECCS with CO_2_ injection) or prioritization of cobenefits to ecosystems and communities (e.g., biochar application in agriculture), other approaches simply focus on the carbon storage as the “product”.^13^ These nonenergy BiCRS pathways generating carbon storage as the only “product” include a range of feedstock forms and storage reservoirs, including dry storage of raw agricultural residues,^16^ forestry residues,^17^ and algae;^18^ anaerobic storage of small diameter wood wastes;^19,20^ burial of biochar;^21^ and injection of bio-oil^22^ or raw biomass^23,24^ (Figure 1).

**Figure 1:**
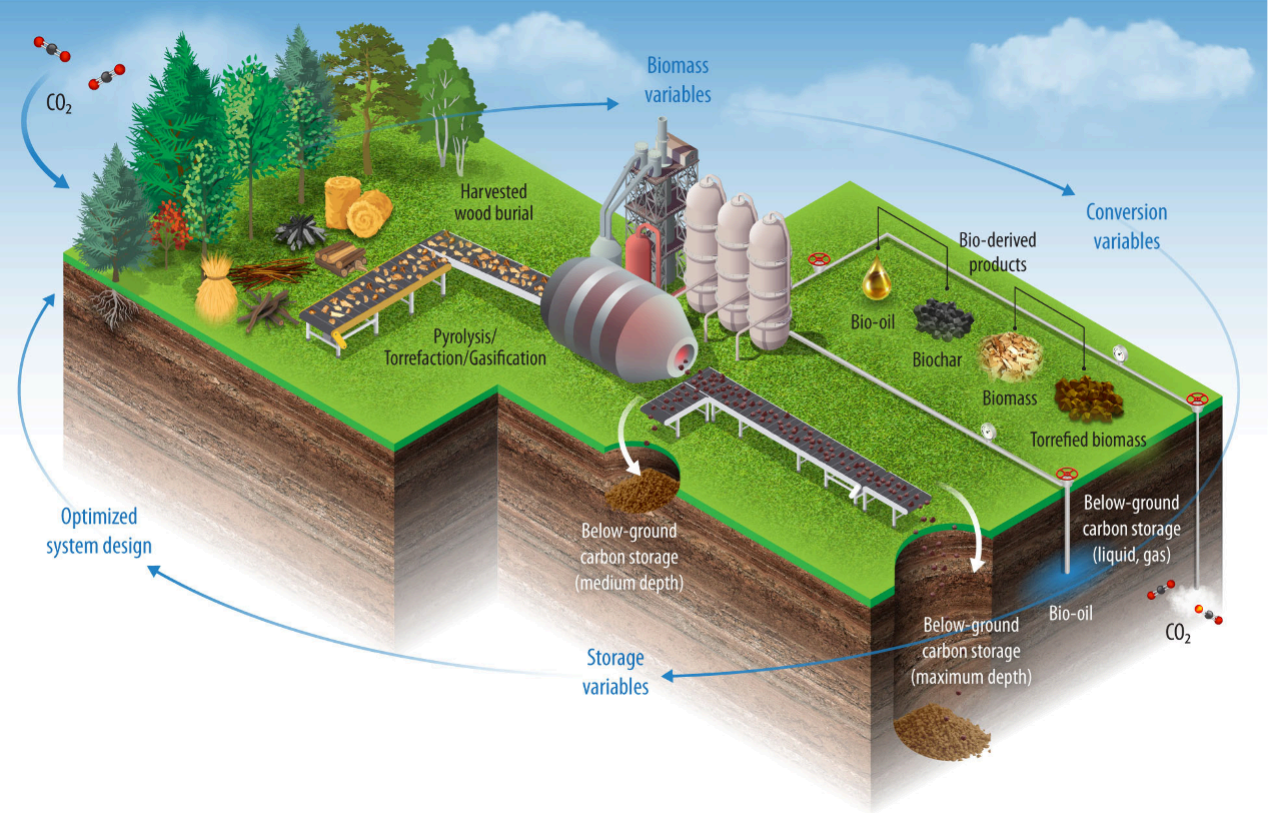
Graphical representation of BiCRS pathways using biomass and bioderived products in various geological scenarios (Illustration by Alfred Hicks, National Laboratory of the Rockies).

Across these nonenergy BiCRS approaches, the durability of carbon products depends upon multiple factors, including composition and chemical recalcitrance of biomass,^25–27^ the conditions of biomass conversion,^28,29^ abiotic conditions or environmental chemistry of storage,^16,30,31^ stability of decomposition products,^32^ biotic decomposition mechanisms (e.g., presence of fungi, bacteria, or other organisms capable of decomposition),^33,34^ and interactions with other materials (e.g., formation of mineral associations, chemical additives).^35^

To address this complex system of interactions, we present a framework for understanding the durability of nonenergy BiCRS pathways organized around two primary axes, the *carbon product form* and the *carbon product fate* (Figure 2). Carbon product “form” is extremely heterogeneous across BiCRS approaches. Durability across carbon product forms can be described by **chemical recalcitrance**, a term encompassing intrinsic molecular structure (e.g., lignin content, aromaticity), degree of condensation, and resistance to enzymatic or oxidative breakdown. Herein, we describe the heterogeneity in chemical recalcitrance across raw carbon products (i.e., **agricultural residues**, **woody biomass**, and **lignocellulosic components of municipal solid waste**, or MSW) and conversion carbon products (i.e., **biochars** and **bio-oils**) (Figure 2A). We exclude gaseous CO_2_ derived from biomass (particularly from BECCS processes), as well as the technological considerations for storing it, which have been extensively reviewed elsewhere (e.g.,^36^).

The carbon storage conditions, or “fates,” are equally as variable as the “forms.” The durability of storage “fates” is a function of the **decay prevention mechanism**, which describes the dominant physical, chemical, or environmental constraint that suppresses biotic or abiotic decay mechanisms (e.g., exclusion of water, oxygen, physical isolation, etc.) (Figure 2B). As part of this review, we focus on opportunities for storage of solid and liquid carbon “products” in terrestrial systems, including **surface storage**, **dry storage**, **anoxic storage**, and **geologic injection**. Durable storage can be achieved through one or multiple decay prevention mechanisms operating in combination. Because these mechanisms map directly onto the pathways by which carbon can be re-exposed to degradation pressures, they also define the primary reversal risks associated with each pathway and the strategies available to mitigate them.

While feedstocks considered in this review can also follow utilization fates (e.g., biochar with agriculture application, mass timber) which may reduce the use of fossil-based feedstocks, be paired with carbon capture and storage, or come with other cobenefits, these are out of scope and have been reviewed elsewhere. In particular, we address surface storage of biochar (which is essentially equivalent to biochar with agricultural application) only briefly here (Section 5.1) as its carbon dynamics and cobenefits have been extensively characterized in prior reviews (e.g.,^37–39^ ), and because its agronomic value frequently warrants treating it as a soil amendment product. We similarly exclude marine storage pathways as processes governing carbon fate in ocean systems and risks to durability are often distinct from terrestrial pathways, and therefore, addressing them would require a separate, domain-specific analysis.

This framework enables a systematic assessment of durability of carbon storage across nonenergy BiCRS pathways. By linking chemical recalcitrance of carbon product forms with the mechanisms of decay prevention associated with different fates, we highlight key assumptions underpinning claims of long-term stability and the potential for reversal. Our goal is to clarify the durability landscape for solid and liquid BiCRS storage pathways, identify key knowns and uncertainties, and outline research priorities needed to advance robust, high-integrity carbon containment.

## 2 Carbon Product Form: Raw Biomass

This review primarily focuses on terrestrial lignocellulosic biomass (agricultural and forestry residues; see Section 2.1–2.2), which is composed mainly of structural carbon components that comprise the majority of plant cell walls: lignin and carbohydrates—including cellulose and hemicellulose.^40^ Most lignocellulosic biomass is approximately 50 wt % carbon (C), 6 wt % hydrogen (H), 40 wt % oxygen (O), less than 2 wt % nitrogen (N), and smaller quantities of other elements including silicon, potassium, calcium, iron, and phosphorus.^41^ Municipal solid waste (MSW) is a potential BiCRS feedstock since it may contain lignocellulosic biomass or other organic waste, but its composition is highly heterogeneous and likely contains nonorganic waste (see Section 2.1.3).

Across BiCRS approaches, biomass composition is a key factor that mediates durability of biomass carbon. Biomass composition is tightly linked to chemical recalcitrance, the resistance of organic materials to deconstruction or decomposition by biotic or abiotic processes, which in turn strongly controls the rate at which carbon can be emitted back into the atmosphere (Figure 2A). However, the composition of biomass varies widely, even within similar feedstocks, and may be impacted by biotic and abiotic processes that occur during growth. Drought,^42^ and pest infestations,^43^ as well as genetics, cultivation, and processing conditions and activities^44^ can all influence biomass composition. Given this intra- and interspecific variability, we describe the key metrics that describe biomass chemical recalcitrance (see Section 2.3) to inform durability under different storage fates. In all cases, clear demonstration of additionality and rigorous, verifiable quantification of counterfactuals are essential for any feedstock used to generate carbon credits (Appendix).

### 2.1 Lignocellulosic Wastes and Residues

#### 2.1.1 Agricultural Residues

Agricultural residues are the byproducts or waste materials left over after the cultivation, harvesting, and processing of crops or livestock. Field residues—including corn stover, rice husks and wheat straw—are typically left behind on the field, used as fodder, burnt, landfilled, or used as a feedstock for bioenergy or bioproducts.^45,46^ Crop process residues consist of the byproduct plant tissues from crops that are otherwise considered waste after harvesting and processing and may include citrus peels, nut shells, molasses, and other components like seeds and roots. Perennial plants such as fruit and nut trees, oilseeds, and dedicated bioenergy crops such as switchgrass and poplar also produce residues during cultivation, harvesting, and processing which include stems and branches, husks, and leaves. Animal residues and waste including manure may also be considered agricultural residues,^45^ but these are out of scope of this review.

The chemical composition of agricultural residues depends heavily on the type of biomass and sourcing process. As with most terrestrial plant biomass, agricultural residues consist primarily of lignin, cellulose, and hemicelluloses that make up the majority of the plant cell walls. However, in comparison to primarily woody feedstocks, agricultural residues may consist of larger quantities of proteins, lipids, inorganics (ash), and extractives including metabolites, free sugars, alkaloids and other components.^47–49^ Agricultural residues composed of leaves, seeds, and shells may have higher N content than wood due to the presence of chlorophyll, alkaloids, proteins, and other N-containing species components.^47–50^ The higher N content, and thus lower C:N ratio, in these feedstocks may render them more susceptible to microbial decay and hence less durable than feedstocks with lower N content.^51^

**Figure 2:**
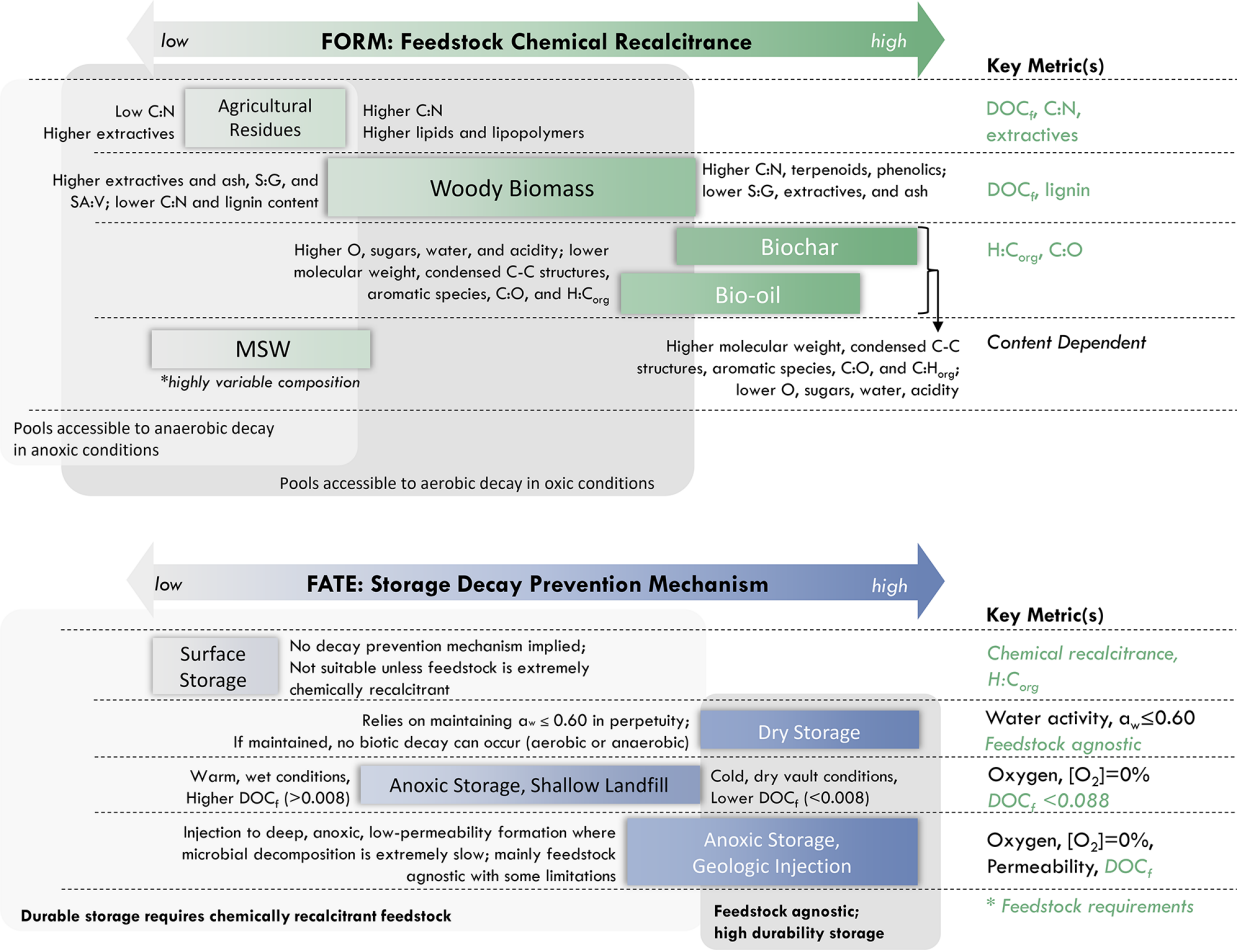
Durability of Biomass Carbon Removal and Storage across feedstock forms (A) and storage fates (B). Feedstock forms (A) range in chemical recalcitrance, which describes their intrinsic ability to resist decay. The range in chemical recalcitrance of each main group of feedstock forms (i.e., agricultural residues, MSW, woody biomass, biochar, and bio-oil) is shown against the gradient from gray to green, with darkest green representing the most chemically recalcitrant feedstock. Characteristics of less or more recalcitrant feedstock within groups are shown on left and right sides of range, respectively. Key metrics describing durability are presented in the column at right. In (B), storage fates are presented along a gradient of decay prevention mechanism (i.e., surface storage, dry storage, anoxic storage shallow landfill, and anoxic storage geologic injection), with gray colors demonstrating less decay prevention and dark blues representing the most complete decay prevention. Descriptions of decay prevention mechanisms are in line with fate and key metrics associated with fates are presented in the column at the right. Across both panels, background gray boxes highlight which pools are available to aerobic and anaerobic decay (A) or require combination with recalcitrant feedstock in (B).

Lipids and lipopolymers may also play essential roles in biomass properties as they relate to microbial decay and durability.^52^ Terpenoids present in forestry residues, waxes present in leaves, and suberin present in bark and roots may occur as recalcitrant components that help improve biomass properties during cultivation leading to more sustainable crops, increased yield, increased soil carbon during biomass cultivation, and increased biomass durability during storage.^52–54^

Agricultural residues typically have higher mineral inorganic or ash content than woody feedstocks—such as shells, bark, and leaves—and retain these components for various purposes including as defense mechanisms and for other vital growth and development functions. These components could represent a relatively larger mass fraction of the biomass relative to the amount of carbon present in comparison to other feedstocks, potentially resulting in lower efficiency as a carbon source for nonenergy BiCRS (higher ash means lower carbon content and hence lower efficiency of moving carbon underground for a given mass of material). Additionally, the presence of inorganics can have significant impacts on thermochemical conversion processes that may be used to convert agricultural residues to bio-oil or biochar.^55^

#### 2.1.2 Forestry Residues

Forestry residues include biomass byproducts or waste materials generated by forestry operations, such as logging, thinning, wood processing, disease and pest mitigation, natural disaster recovery or risk reduction, and invasive species projects. These residues are typically nonmerchantable components of trees and other vegetation and can remain on-site in the forest, be piled and burned, or be collected for alternative uses.^56^ Forestry residues exhibit distinct chemical compositions that influence their stability in different storage reservoirs.

Chemical composition of forestry residues varies significantly among hardwoods (angiosperms) and softwoods (gymnosperms). Hardwoods are comprised of 38–49 wt % cellulose, but generally lower lignin (20–30 wt %) than softwoods (26–34 wt %^57^). Softwoods have less noncellulosic polysaccharides (7–14 wt %) than hardwoods (19–26 wt %), but there is a great deal of variation across softwoods, hardwoods, and mixed woods (an intermediate classification).^57,58^ In addition to structural biopolymers, wood contains a minor fraction of organic compounds, including polyphenols (e.g., stilbenoids, flavonoids, tannins) and terpenoids.^59^ These extractives typically account for 5–10 wt % of the dry biomass but can reach up to 20 wt % in certain tropical and subtropical species.^58,60^ In terms of chemical recalcitrance, softwoods do not naturally contain syringyl (S) lignin and therefore are composed primarily of guaiacyl-rich (G) lignin that is more condensed and chemically recalcitrant (e.g.,^61^). Hardwoods typically have higher S:G ratios ranging from ∼1–3 which results in more linear and less cross-linked lignin that is easier to degrade (see Section 2.3 for additional information on durability metrics).

At the tree-level, forestry residues are comprised of sawdust and shavings, leaves and needles, bark, branches, and tree boles—all of which have varied chemical properties. For example, needles are rich in extractives (27 wt %),^62^ including resins, waxes, and phenolics. They decompose more readily than some other woody components due to their elevated N (1.2 wt %)^63^ and ash content. Moderate to high levels of lignin (24–35 wt %) and cellulose (38–41 wt %)^62,64^ provide some structural integrity, but empirical-measured rates of decay suggest a rapid 2.7-year half-life for needles.^65^

Small branches and twigs, which include a mix of small-diameter wood and bark, have greater structural stability than needles but still retain moderate levels of extractives and hemicellulose, which influence their decomposition rates.^66^ With higher lignin and cellulose content than needles, branches exhibit improved resistance to microbial and oxidative degradation. However, their moderate hemicellulose levels make them susceptible to moisture uptake, which can affect their stability in storage. The presence of bark-bound tannins, lipids (i.e., terpenes), and phenolics can contribute to long-term chemical stability due to their condensed structures, antioxidant, antimicrobial, and preservative properties,^53^ but also influence interactions with environmental conditions over time.

Finally, boles or stemwood, which represent the most chemically recalcitrant component, are characterized by relatively high cellulose and lignin content with low extractives and ash. This composition, in addition to lower surface area to volume ratio, enhances their resistance to microbial decay and makes them the most stable for long-term storage.^67^ The low surface area to volume ratio of boles compared to finer residues such as needles and branches also slows decomposition kinetics, as microbial colonization is limited by reduced oxygen diffusion and lower relative exposure of reactive surface functional groups.^68–70^ Additionally, the reduced presence of water-soluble organics and lower nitrogen content diminish their nutritional attractiveness to decomposers.^71^ For these reasons, bole-derived biomass is generally considered the most recalcitrant and stable form of raw biomass in both natural decay pathways and storage reservoirs.

#### 2.1.3 Municipal Solid Waste

Municipal solid waste (MSW) is the residential and commercial waste that is typically collected for landfilling. The main components of MSW are food waste, paper products, plastics, textiles, glass, and metals. Analyses of the amounts of each component are difficult to conduct and vary substantially by location and time of year. For example, in the United States, in winter, there are increases in wrapping paper and cardboard while summer and fall have increases in yard waste.^72^ Analyses from the United Kingdom spanning 1992 until 2004, show the amount of food waste ranged from 20 to 44 wt %, paper and cardboard from 22 to 26 wt %, glass 5–9 wt %, textiles 1–3 wt %, ferrous metals 3–6 wt %, nonferrous metals 1–2 wt %, diapers 3–7 wt %, and fines 1–9 wt %.^73^

An analysis of MSW in the United States in 2010 found the following compositions prior to recycling: paper and cardboard (28.5 wt %), food waste (13.9 wt %), yard waste (13.4 wt %), plastics (12.4 wt %), metals (9 wt %), rubber leather and textiles (8.4 wt %), wood (6.4 wt %), glass (4.6 wt %), and other (3.4 wt %).^74^ Ferrous metals may be removed with magnets before any processing. More recently, artificial intelligence (AI) sorting technology has improved to remove more components for recycling.^75^ MSW is generally amenable to high-temperature, destructive conversion technologies to produce biochar, mineral-rich ash, and gases from processes like gasification.^76^ However, given the significant variability in composition across spatiotemporal gradients, we focus the majority of this review on the products and processes associated with lignocellulosic biomass, and not the other components of MSW.

### 2.2 Dedicated Crops

A dedicated or purpose-grown crop is a nonfood crop grown for a specific purpose and usually refers to crops grown for bioenergy purposes (i.e., switchgrass). At this time, dedicated crops that would be primarily associated with BiCRS may also generate energy as a coproduct (BECCS). While dedicated crops are already used in bioenergy systems, their application in BiCRS and carbon crediting frameworks remains limited and subject to ongoing debate. Key challenges include demonstrating additionality, ensuring sustainable biomass sourcing, minimizing land use change impacts, defining appropriate counterfactual baselines, avoiding market leakage, and accounting for emissions associated with cultivation (Appendix). Should dedicated carbon crops be accepted for use in BiCRS pathways, they would need to address such issues. Of course, it is worth noting that all feedstocks used for BiCRS approaches should similarly meet criteria describing high quality CDR, such as rigorous counterfactual assessment and sustainable feedstock sourcing.

Potential dedicated carbon crops may include herbaceous perennials such as switchgrass and miscanthus, woody perennials such as poplar, willow, and pine, and other crops such as sorghum.^15^ Pett-Ridge et al. (2023) thoroughly describe analyses and potential scenarios using perennial carbon crops to identify opportunities and challenges associated with their use in BiCRS pathways. Meanwhile, land conservation innovators are now developing and piloting perennial mixed-species grain plantings, native prairie-grass systems, and grain-producing perennials that also produce biomass without the need for regular tillage (e.g.,^77^). These systems could be considered a new category of dedicated cropping approaches, which would be designed around system resilience, climate adaptation, and the development of soil microbial communities that contribute to efficient soil carbon retention in order to meet biomass sourcing criteria for high-quality CDR.

In the near term, there are a wide range of waste biomass feedstocks that can be sustainably used for BiCRS, including agricultural wastes, forestry residues, and municipal solid waste.^13,15^ For the long-term, sustainable practices associated with using dedicated crops need to be broadly developed or proven and deployed to avoid potential negative impacts on the environment, society, and the market. In the ensuing sections, we therefore focus on chemical recalcitrance of waste biomass available in the near-term.

### 2.3 Durability Metrics for Raw Biomass

When assessing raw biomass chemical recalcitrance, the carbon-to-nitrogen ratio (C:N) is a fundamental property of biomass that influences its decomposition rate and microbial accessibility (e.g.,^71,78^ ). A higher C:N typically signals a greater proportion of structural carbon relative to nitrogenous compounds and is associated with slower microbial degradation.^51,67^ In contrast, low C:N materials decompose rapidly, often accelerating the breakdown of more recalcitrant substrates through cometabolism.^25^ Further, removal of high N wastes or residues from native environments may have significant implications for nutrient cycling and future fertilizer use (i.e., potential for increased downstream emissions if removed^79^). C:N varies widely among biomass types—ranging from <15 in microalgae^80^ and manures^81^ to 100–500 or higher in woody biomass.^82,83^ This variation in C:N and the resulting chemical recalcitrance has implications for which forms of raw biomass are suitable for nonenergy BiCRS approaches.

The relative abundance and architecture of the different components of the plant cell wall may vary and may also impact the chemical recalcitrance of the biomass^84^ (Figure 3). Among the structural carbon components, lignin is especially recalcitrant.^85^ Lignin is a complex aromatic polymer in the plant cell wall that is responsible for structural rigidity and hydrophobicity.^86^ It is composed primarily of three monolignols: coniferyl alcohol (G units, guaiacyl), sinapyl alcohol (S units, syringyl), and p-coumaryl alcohol (H units, p-hydroxyphenyl).^87,88^ The relative abundance of these monomers, and particularly the S:G ratio, has been proposed as a determinant of reactivity (e.g.,^89^ but see^90^). Lignin-rich biomass types with low S:G ratios, such as coniferous trees, tend to resist microbial decay and oxidation, making them more suitable for long-term storage applications, such as anoxic storage (see Section 5.2). In contrast, biomass with less lignin (i.e., leaf-rich agricultural residues) or high S:G ratios (such as hardwood tree species), generally degrade more rapidly, although decay rate is additionally mediated by climatic conditions, such as mean annual temperature and actual evapotranspiration.^91,92^ Nonetheless, metrics such as total lignin content, the lignin-to-cellulose ratio, and the extent of polymer cross-linking often inform assessments of carbon durability (^89^ and citations within).

**Figure 3:**
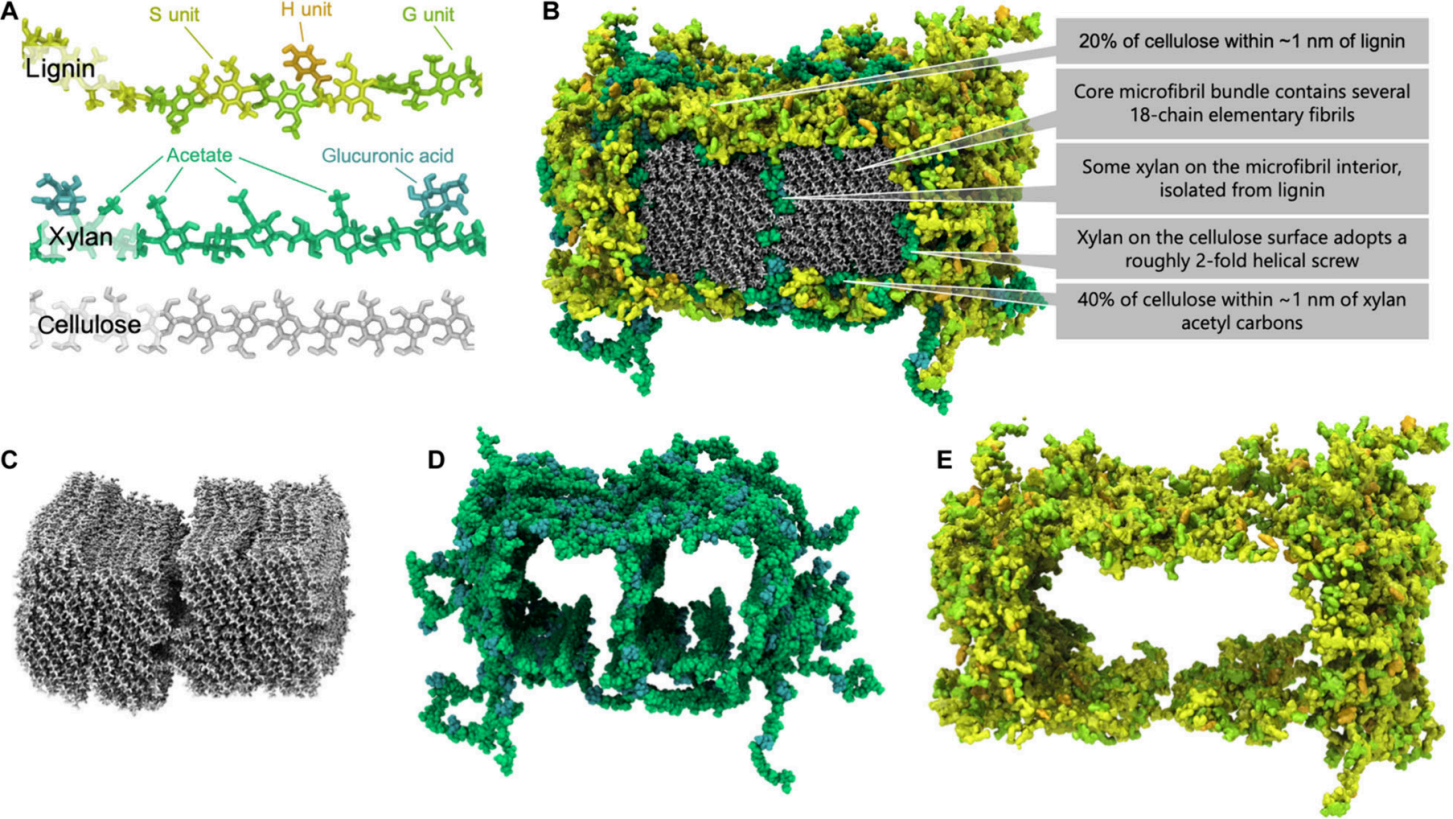
Directly Reproduced from (Addison et al., 2024).^84^ Copyright 2024, The American Association for the Advancement of Science. Experimentally informed atomistic model of poplar secondary cell wall showing (A) individual biopolymer constituents and (B) macromolecular assembly of the primary biopolymers present in biomass: lignin, cellulose, hemicellulose and xylan. Cellulosic core bundles (C) are contained within a sheath formed by hemicellulosic components (plus xylan bundles) (D), which provide a barrier between lignin (E) and cellulose. These polymers vary in structure and composition based on many factors, which may ultimately impact biomass recalcitrance toward biotic and abiotic decomposition and deconstruction, and hence biomass storage durability.

## 3 Carbon Product Form: Thermochemically Converted Biomass

Raw biomass can be converted or stabilized by heating processes to produce condensed, recalcitrant, and carbon-rich products, particularly biochar and bio-oil (Section 3.1). These thermochemical conversion processes have different names depending on the temperature, heating rate, and other factors (Section 3.2; Figure 4). Different reactor designs and process conditions lead to different yields of products, properties, and emission profiles.^93,94^ Durability of conversion products similarly depends on initial feedstock composition, reaction processes, and storage reservoirs—and can be estimated based upon initial durability metrics, such as the molar ratio of hydrogen to organic carbon (H:C_org_)^37^ and potentially also random reflectance (R_o_)^32^ (Section 3.3), although recent critiques of R_o_ highlight that such metrics are not intended to classify resistance to biological decay.^95^

**Figure 4:**
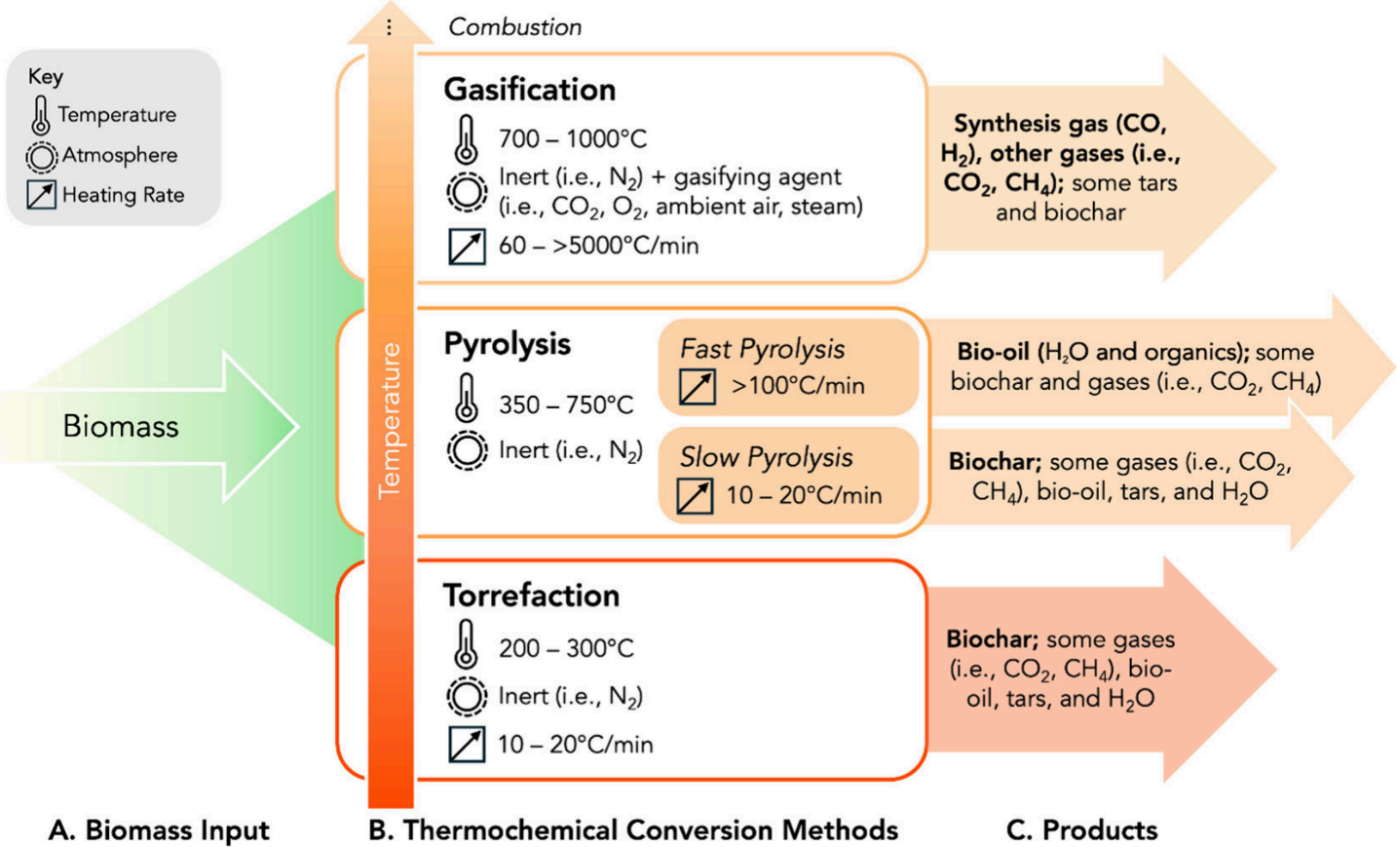
Summary of biomass thermochemical conversion processes used to produce major bioproducts for carbon storage within the scope of this work, including pyrolysis, torrefaction, and gasification. Minor nitrogen- and sulfur-containing species (e.g., NH_3_, HCN, H_2_S, COS, trace heterocyclic compounds) are not shown due to their low abundance in lignocellulosic biomass.

### 3.1 Thermochemical Conversion Products

#### 3.1.1 Biochar

Biochar is a carbon-rich, solid coproduct of thermochemical biomass conversion, primarily formed during torrefaction and pyrolysis processes. While the term biochar is often associated with agricultural soil amendment, for BiCRS purposes, it broadly refers to any solid char product produced under oxygen-limited thermal treatment and intended for long-term carbon storage. The defining chemical characteristic of biochar is its high aromatic carbon content, resulting from the progressive dehydration, depolymerization, and recombination of lignocellulosic polymers into fused aromatic ring structures.^96^ This condensed aromaticity confers chemical recalcitrance, low bioavailability, and resistance to microbial degradation—key properties for durable carbon sequestration.^21,32^ Additionally, the carbon yield of biochar can be typically on the order of approximately 50%, depending on the feedstock and conditions, meaning it could be a relatively efficient approach to durable carbon sequestration.^97^ However, the specific elemental composition, ash content, surface area, porosity, and other important chemical and physical properties of biochar vary widely depending on feedstock type, peak pyrolysis temperature, heating rate, and residence time.^28^

#### 3.1.2 Bio-oil

Bio-oils are complex mixtures of thermally degraded biomass components produced primarily during fast pyrolysis at moderate to high temperatures (350–600 °C). Unlike biochar, bio-oils are liquid-phase products consisting of hundreds of oxygenated organic compounds, including acids, aldehydes, ketones, phenolics, and anhydrosugars, along with varying amounts of water and solid particles.^98^ Their dark, viscous nature and high oxygen content (typically 35–50 wt %) distinguish them from fossil-derived fuels and complicate their long-term stability. For BiCRS applications, however, bio-oils are not necessarily viewed as fuel intermediates but as carbon-rich liquids that can be stored in anoxic or engineered containment systems, potentially offering durable sequestration if stabilized properly.^22^

The viscosity, acidity (pH ∼ 2–3), and chemical instability of bio-oils pose challenges for long-term storage in standard conditions, as continued polymerization and phase separation may occur over time, particularly under warm or aerobic conditions.^99^ These phases consist primarily of an aqueous phase containing oxygenated hydrocarbons (commonly referred to as “wood vinegar” due to the presence of acetic acid) and a viscous organic fraction consisting of heavier “tar” components and lighter “oil” components. However, these aging properties can be advantageous for durability claims in geologic systems (e.g., aromatization increases condensed fraction; see following sections for discussion). Additionally, the carbon yield in bio-oil can reach over 30% of the initial biomass carbon depending on feedstock and reactor conditions, making it a valuable target for BiCRS when durable containment options are available.^22^ As with biochar, the effectiveness of bio-oil as a BiCRS pathway depends on matching its chemical and physical properties with appropriate containment environments and evaluating storage permanence over centennial to millennial time scales.^100,101^

### 3.2 Thermochemical Conversion Processes

#### 3.2.1 Pyrolysis and Torrefaction

Pyrolysis refers to the thermal degradation of a feedstock (e.g., lignocellulosic biomass) in an oxygen-deficient atmosphere to a maximum temperature of approximately of 200–750 °C. Pyrolysis processes can be fast or slow (i.e., 10 °C/min for slow, up to approximately >250 °C/sec for fast), encompass many types of reactor configurations (e.g., microwave, fluidized beds), and are typically conducted to favor the formation of liquid and/or solid products. Typically, fast pyrolysis is used to generate liquid products in the range of 30–60 wt % yield with the remaining yield being char (varies, typically on the order of ∼ 20 wt %), and noncondensable or permanent gases (up to ∼ 15 wt %) depending on reactor parameters and feedstock used.^98,102–104^ Pyrolysis liquids (oil, aqueous fractions, tar, etc.) are typically recovered through condensation systems while biochar and ash are recovered via a variety of mechanisms depending on the reactor configuration. Gaseous products may be exhausted, flared, recycled or combusted.

Torrefaction is a term commonly used to refer to pyrolysis performed at a relatively slow rate (i.e., 10–20 °C/min) and to a maximum temperature of approximately 200–300 °C.^105^ This process is used to favor the formation of solid, biochar products that could be used for environmental, agricultural, energy and/or materials applications (e.g.,^106^). The yield of char is typically on the order of 35–50 wt % while gas and liquids each make up the remaining yield of the products.^98^ The properties of biochar may vary as a function of pyrolysis temperature (i.e., increases in temperature may lead to reduced surface area, reduced volatile matter, and lower H:C_org_), though some properties may not be as clearly temperature-dependent (i.e., carbon content)^107^ (see Section 3.2.3 for additional information).

#### 3.2.2 Gasification

Gasification refers to heating feedstocks at high temperatures (typically 700–1000 °C) in a mixture of inert gas and gasifying agent, which can be CO_2_, ambient air, oxygen (O_2_), or steam, to produce synthesis gas—a mixture of carbon monoxide (CO) and hydrogen gas (H_2_)^108^—which can then be combusted to produce power or used for the synthesis of products (e.g., ammonia, hydrogen, methanol, or hydrocarbons liquid fuels).^109^ Gasification can be achieved in a variety of reactor configurations (fixed bed [downdraft or updraft], fluidized bed, entrained flow) and with or without a catalyst to increase yields of the desired product, synthesis gas, and decrease byproducts which include biochar, CO_2_, H_2_O, CH_4_, and tars (e.g., naphthalene).^110^

A water–gas shift catalyst and steam can be used to further convert the product gases to higher yields of CO_2_ and H_2_, at the expense of CO, which is desirable if the selected product is H_2_ only and the CO_2_ can be captured for sequestration. The CO_2_ yield of gasification depends on the desired H_2_:CO ratio the gasifier was designed for and can be as little as 7.5 volume percent of the gas produced^111^ or as high as nearly 100% of the biomass carbon when the desired product is H_2_ alone and a water–gas shift catalyst is used to convert CO to CO_2_.^112,113^ It is common for the gasification biochar to be recirculated through the reactor to increase gas yields or removed and combusted for heat, but it can also be used for carbon sequestration via soil amendment or burial. Typical biochar yields from biomass gasification range from less than 1- 14 wt % of the biomass fed and the ash content of the biochar varies widely, at least from 4 to 73 wt %.^114,115^ Increasing gasification temperature and decreasing biomass particle size both decrease biochar yields.

#### 3.2.3 Conditions Mediating Thermochemical Conversion Processes

The conditions of thermochemical conversion processes have substantial impacts on the carbon yield and product distribution and properties, as well as the associated emission profiles. Generally, higher temperatures result in significant cracking, dehydration, dehydrogenation, condensation, and other reactions that lead to higher yields of gases and liquids and lower yields of carbon-rich solids.^116^ Torrefaction—“low and slow” pyrolysis (i.e., low temperature with a slow heating rate)—results in significant condensation and carbonization of biomass, thereby producing higher yields of biochar.^117,118^ Slow pyrolysis, while conducted at higher temperatures than torrefaction, also results primarily in carbon-rich biochar. Biochar, produced in lower yields from fast pyrolysis, is also highly condensed carbon produced from the loss of chemical functional groups including oxygen.^93,96,119^ Fast pyrolysis liquid products may also vary depending on the temperature, range from ∼ 15–30 wt % water and 50–60 wt % carbon content, and vary in chemical constituents, viscosity, density, and other properties as well.^119–122^ Biochar produced from gasification processes conducted at higher temperatures and in the presence of oxygen may consist of lower carbon content and higher composition of ash and inorganic constituents. Biochar from gasification is also produced in lower abundance relative to gases or liquids/tars.^123,124^

The residence time and heating rates of the feedstock and vapors generated during thermal decomposition can also impact the yields and properties of the products. For example, longer residence times at lower temperatures generally result in the occurrence of secondary reactions including dehydration, cracking, and condensation that may lead to higher yields of solids and gases as well as increased production of water as a product.^125^ Heating rates controlled by the reactor conditions may also impact the yields and product properties. Higher heating rates typically lead to increased yields in liquids and gases while lower heating rates produce primarily biochar.^126^ Feedstocks and reactors will also have differences in heat transfer efficiencies, which may also lead to differences in product distributions and properties. Generally, higher heat transfer efficiency, like higher heating rates, can enhance oil and gas production.^125^

The properties, including composition, of the biomass feedstocks can also play a major role in the carbon yield, emissions profiles, product distribution, properties and efficiency of thermochemical conversion processes. Biomass such as grasses (i.e., switchgrass, miscanthus) and agricultural residues may have higher inorganic or ash content in comparison to forestry or wood products (i.e., >10 wt % for residues such as corn stalk in comparison to ∼ 1 wt % of ash in woody residues).^38,127^ The presence of inorganics and ash in the feedstock may result in higher ash content, and therefore lower carbon content, in solid product yields as well as higher water content in liquid yields (and lower organic, oil yields) from thermochemical conversion processes such as fast pyrolysis.^55,127^ Inorganics also catalyze various cracking, water–gas shift, and other reactions that can lead to higher production of gas products including syngas.^128,129^

Nitrogen and sulfur present in the feedstock may also impact the conversion process as well as the composition and properties of the resulting solid, liquid and gaseous products. For example, feedstocks with relatively high N content (>5 wt %) may produce higher yields of gases containing N including NH_3_ and HCN, tars or liquids containing amines, amides, nitriles, pyrroles, and biochars also containing residual nitrogenous species than those produced from lower-N feedstocks such as wood.^127,130^ The relative composition of biopolymers such as lignin and carbohydrates may also impact product properties upon thermochemical conversion processes. For example, feedstocks with higher lignin contents may lead to pyrolysis oils with higher average molecular weight and therefore differences in aging properties,^128,131^ which may impact durability.

Lastly, it is important to consider the multivariate relationships that may occur among the various biomass properties and characteristics that could impact the properties of the products from thermochemical conversion processes, particularly as some biomass show distinct correlations among different compositional features (Figure 5).^38,132^

**Figure 5:**
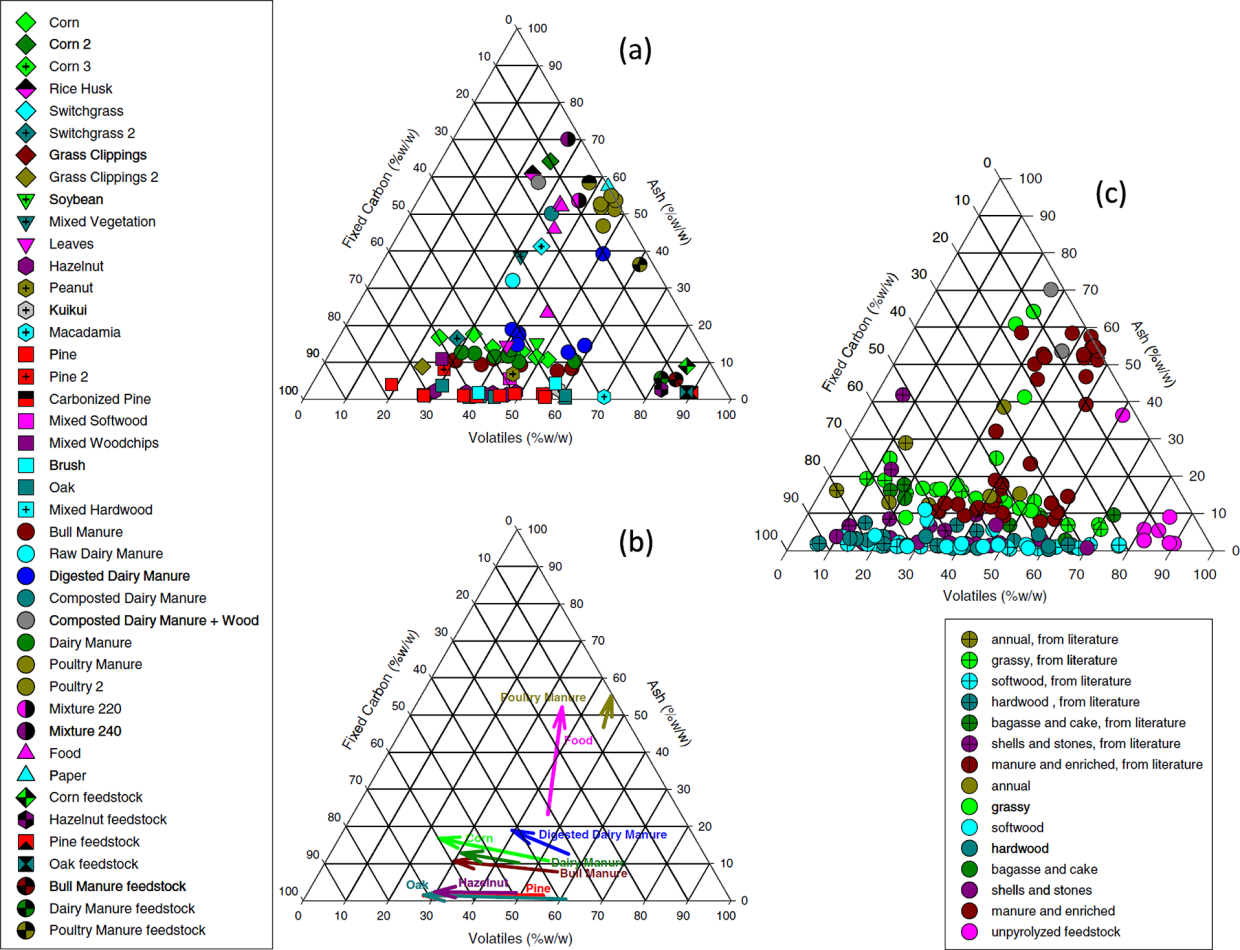
Directly reproduced from (Enders et al., 2012).^38^ Copyright 2012, Elsevier. Triangle plots show (A) the relationships among ash, volatile matter and fixed carbon contents; (B) the effect of thermochemical conversion temperature (arrows connect biochars of the same feedstock pyrolyzed at 300 and 600 °C); and (C) a comparison to literature data.

### 3.3 Durability Metrics for Conversion Products

#### 3.3.1 Biochar Durability Metrics

There are many different chemical and physical properties of bio-oil and biochar products that may impact the chemical recalcitrance of these products when considered separately from or in conjunction with their storage conditions.^133,134^ Many analytical techniques including ultimate analysis and carbon structure analyses can be used to assess stability.^135^ The molar ratio of hydrogen to organic carbon (hereafter, H:C_org_) of biochar has been considered the most important proxy for chemical recalcitrance.^136^ The H:C_org_ is an indication of the abundance of recalcitrant, condensed carbon species, where lower values (i.e., <0.5) would be representative of longer-term durability.^137^ Similarly, low O:C ratios are also indicative of lower oxygenated hydrocarbons capable of decomposing and values <0.2 are considered more durable biochar (though this metric shows less significant correlation to durability relative to H:C_org_).^37,138^ Spectroscopy including NMR and Fourier transform infrared spectroscopy (FTIR) can be used to assess aromaticity and the degree of aromatic condensation.^135^

Biochar durability when buried under soil could also be assessed based on traits that demonstrate biochar similarity to coal, which is known to be stable in geological formations for millions of years. The random reflectance, R_o_, of biochar is measured via microscopy and is also considered a proxy for biochar stability.^32^ For biochar to be considered highly durable, it needs to have R_o_ > 2%, classifying it as the inert maceral “inertinite.” Random reflectance has been used in organic petrology applications to measure the degree of aromaticity and condensation in organic materials where higher aromaticity produces higher random reflectance values.^139^ While this is a standard method for petrology applications, there may be challenges with using this metric for biochar durability claims (e.g.,^95^). Many R_o_ measurements need to be made of prepared samples to accurately capture representative values and additional experimental data (i.e., long-term monitoring data from field sites) may be needed to support long-term durability claims of biochar with R_o_ > 2%.

In addition to H:C_org_ and R_o_, there are other chemical constituents that influence the chemical recalcitrance of a biochar product. The presence of inorganics, nitrogen, and ash in biochar and its pH may directly impact the reactivity properties (i.e., liming effect whereby biochar may impact the pH of surrounding soil, potentially impacting emissions or carbon mineralization processes) of biochar in the presence of certain species, and especially in agronomic applications.^136^ While the presence of these components in biochar may otherwise offset the total amount of durable carbon present in the biochar (i.e., the more ash present in biomass, the less the mass fraction of carbon), it has not been demonstrated whether these components directly impact the durability of the carbon in a geological storage setting either positively (synergistically) or negatively (through promotion of decomposition, aging, etc.). However, at least one study has shown that low ash biochar is potentially better suited than high ash biochar for biospheric storage based on observed increases in native soil organic carbon mineralization during incubation studies.^140^ Additionally, biochar may also contain residual low molecular weight leachable compounds and sugar species from biomass that could decompose and reduce the durability of biochar if exposed to biotic and abiotic processes.

Finally, physical properties of the biochar—including particle size, surface area, and porosity—can influence the durability of the biochar directly, or indirectly through interactions with storage media (i.e., soil). For example, smaller particle sizes with higher surface areas may be more susceptible to physical leakage or transport (e.g., aeolian transport;^141,142^ or in water^143,144^ ). Similarly, high-porosity biochar may have lower mechanical strength, making it more susceptible to weathering processes.^145^

#### 3.3.2 Bio-oil Durability Metrics

Chemical recalcitrance of bio-oils is less studied than for raw biomass and biochar. Chemical recalcitrance of bio-oil in geological storage scenarios may be related to the total carbon content, the functional groups present, and the presence of sugars and other low molecular weight hydrocarbons. However, no peer-reviewed studies have been conducted to demonstrate relationships between bio-oil properties and long-term storage durability. Based on known stability properties of pyrolysis oils aboveground, we provide some hypotheses and perspectives to consider for studying pyrolysis-oil durability stored in geologic reservoirs or formations,^146,147^ as this is the only storage fate considered for bio-oils. We urge caution and additional research before widescale adoption or application of these preliminary metrics.

At the molecular level (and similar to biochar and raw biomass), we hypothesize that bio-oils with larger molecular weight and higher condensation of the carbon species present may contribute to chemical recalcitrance or stability as these characteristics are typically associated with greater aromaticity, lower volatility, and reduced biodegradability. In subsurface environments, such molecular structures are less prone to microbial metabolism and chemical alteration, particularly under anoxic, high-pressure, and thermally stable conditions.^22^ The presence of functional groups such as carbonyls, alcohols, acids, and aldehydes, and components such as free sugars, may reduce the bio-oil stability and render it biodegradable under some conditions, whether during temporary storage or potentially during storage in geological formations depending on storage conditions.^148,149^ Oxygenates in bio-oil also contribute to differences in pH; the presence of acids and low bio-oil pH may introduce risks to geologic storage stability as it may increase bio-oil reactivity with surrounding rock or media, potentially enabling migration or leakage.

At the bulk or macroscopic level, several chemical properties of bio-oils influence the stability or integrity of the decay prevention mechanism (i.e., anoxic geologic injection). Therefore, while not necessarily direct measures of chemical recalcitrance, we include a brief discussion of these indirect metrics here. First, we hypothesize that density and viscosity will be key mediators of bio-oil stability in geologic formations. For example, if bio-oil is denser than brine, it is less likely to undergo upward migration during geological storage, rendering denser bio-oils potentially more durable than less dense bio-oils. Viscosity may also help improve bio-oil durability as more viscous bio-oils may also be less likely to migrate after well injection.

A final unknown surrounding bio-oil chemical recalcitrance and durability in geologic formations is phase separation. Bio-oil is known to consist of significant amounts of water (on the order of 20 wt %) and to phase separate into two (or more) distinct liquid phases typically over time or under specific temperature, moisture, or pH conditions, and particularly in aqueous salt solutions.^150,151^ As bio-oil is injected into saline aquifers, it has a propensity to phase separate into two main phases: (1) the aqueous phase, with is rich in low-molecular-weight, water-soluble compounds (acids, aldehydes, ketones, alcohols, and sugars), contains a large fraction of the polar, reactive oxygenates, and is considered less stable in subsurface environments due to solubility, low viscosity, and microbial accessibility; and (2) the organic phase, which contains larger, more hydrophobic molecules (phenols, furans, anhydrosugars, lignin-derived oligomers, and heavy aromatic structures) and has higher carbon content and molecular weight. The organic phase is more viscous, less polar, and less water-soluble, and is considered more chemically recalcitrant due to lower reactivity and poorer solubility in formation waters.^98,152^ The relative proportion of carbon across these phases is an important frontier for future research and understanding both chemical recalcitrance and durability under geologic injection fates.

## 4 Carbon Product Fates: Mechanisms of Decay

Carbon contained within biomass residues and wastes not used in BiCRS pathways is generally returned to the atmosphere via decomposition or fire. The natural processes of decomposition to CO_2_, CH_4_ and other products are especially relevant to understand in order to assess likely pathways of reversal for nonenergy BiCRS pathways. For example, the rate of decomposition and abundance of products generated primarily depends on the feedstock, microbial degrading consortia present, and abiotic conditions (temperature, light, moisture, fire, etc.).^153^ It is essential to understand how biomass and bioproducts otherwise would have decomposed because these same mechanisms are what need to be mitigated to prevent reversals during a storage fate. We therefore discuss biotic (Section 4.1) and abiotic (Section 4.2) pathways of decay in the following section.

### 4.1 Biotic Mechanisms

In the absence of biomass storage in biologic or geologic reservoirs or in products, biomass will degrade at end of life. Natural decay processes represent significant risks to the durability of BiCRS storage pathways, especially those utilizing raw biomass. In natural contexts, fungi and bacteria are the major microbial decomposers of biomass. Fungi include white and brown rots (Basidiomycota) and the less well-studied soft rots (for the most part, Ascomycota). Bacteria are omnipresent in decomposing wood, with some species able to survive fully independently, while others depend on the byproducts of fungal degradation to survive.^154^ Wood-decomposing bacteria and fungi can have symbiotic or mutually inhibitory relationships.

Downed and deadwood provides a useful case study for the processes of decomposition in woody biomass.^155^ Decomposition of deadwood is predominantly restricted to fungal phylum Basidiomycota class Agaricomycetes. Structures that are rich in lignin or pure lignin, such as those left behind by brown or soft rots, can become incorporated into recalcitrant complexes that are difficult for microbes to degrade rapidly. Fresh deadwood has a low nitrogen content of 0.03–0.19% by dry mass^156^ which is a major limitation for decomposition, as described in Section 2.1. The proportion of N increases during decomposition, because C is lost to mineralization, microbes fix new N, and fungi import N via mycorrhizae. N fixation by microbes is energy-intensive, and fresh deadwood contains an appropriate energy source for this process: easy-to-access carbohydrates. N fixation proceeds at an elevated rate in fresh wood, compared with more decomposed wood or soils. Storage fates for biomass should be designed with consideration of biotic decay mechanisms in order to limit or prevent re-emission of stored carbon. We describe specific mechanisms in more detail below.

#### 4.1.1 Brown Rot

Brown rots rapidly degrade nearly all cellular components, leaving behind modified lignin. They do not rely solely on enzymes for decomposition. During mycelial growth, brown rots secrete oxalic acid— (COOH)_2_—and generate radical oxygen species (chelator-mediated Fenton chemistry) to attack and break down cellulose and hemicellulose.^157^ Iron reduction is an indication of ongoing Fenton chemistry. Some brown rots carry genes to produce secondary metabolites which serve as extracellular Fe^3+^ reductants, which drives the Fenton reaction and further helps brown rots decompose cellulose.^158^ While they cannot alter the structure of lignin, brown rots can generate recalcitrant particulate lignin. One consequence of this in nature is that brown rots may increase carbon stocks in forest soils (unlike white rots, which mineralize lignin completely).^33^

#### 4.1.2 White Rot

In contrast, white rots use specific enzymes to decompose all wood components, including lignin. Cellulose is gradually degraded, while lignin is completely mineralized.^27^ Some white rots can reduce iron and drive Fenton chemistry, but to a much lesser degree than brown rots. White rots do not secrete any oxalic acid and can even produce oxalic-acid-degrading enzymes to eliminate excess oxalic acid in their growing environments, counteracting and directly competing with brown rots. White rots produce several major peroxidase enzymes which degrade lignin, including lignin-attacking oxidoreductases such as manganese peroxidase, lignin peroxidase, and versatile peroxidase, which are high oxidation potential Class II peroxidases. Other lignin-degrading enzymes include dye-decolorizing peroxidases and laccases.^159^ To degrade crystalline cellulose, white rots produce cellobiohydrolases and lytic polysaccharide monooxygenases.

#### 4.1.3 Soft Rot

Historically, soft rots were characterized as stress-tolerant fungi that inhabit extreme environments such as polar regions, deserts, and wet tropical forests. However, recent evidence suggest that they are abundant across less extreme environments, including temperate forests.^160^ Not all soft rots are deadwood decomposers. Some are pathogens or endophytes. Soft rots, like brown rots, can generate recalcitrant particulate lignin because they cannot directly degrade lignin. They use wood cell wall erosion to access carbohydrates. In general, soft rots attack hardwoods to a greater extent than softwoods. In hardwoods, the carbohydrate fraction is removed faster than the lignin while in softwoods, lignin is removed faster than cellulose or hemicellulose.^161^

#### 4.1.4 Bacteria

Bacteria exist in both terrestrial and aquatic environments. They can invade hardwoods and softwoods, including woods that are typically considered decay-resistant and chemically treated wood. Along with Basidiomycota fungi, certain Gram-negative aerobic bacteria are capable of degrading lignin.^162^ Unlike white rots which require carbohydrate cosubstrates, certain bacteria can use lignin as a sole carbon and energy source. However, while these microbes can change the structure of lignin, they do not necessarily oxidize it all the way to CO_2_; more studies are needed to characterize this pathway. Certain bacteria may be more effective at degrading lignin in high-pH conditions (e.g., pH 8) as compared with white rots which are most effective at pH 3.5–6.^163^ Bacterial decomposition primarily affects sapwood, but bacteria also degrade ray parenchyma and pit membranes in the heartwood. Some bacteria can both fix N and degrade complex biopolymers like cellulose, making them independent deadwood decomposers. However, most depend on products of wood decomposition by other decomposers to subsist.

#### 4.1.5 Methanogens

In anaerobic environments, methanogenesis represents the terminal step of a tightly coupled microbial cascade in which several trophic groups cooperate to degrade organic matter. Anaerobic decay begins with hydrolytic bacteria that depolymerize complex biopolymers into soluble sugars, amino acids, and long-chain carboxylic acids. These products then undergo acidogenesis, in which fermentative microorganisms convert them into short-chain carboxylic acids, ammonia, CO_2_, and hydrogen (H_2_). Acetogenic bacteria subsequently oxidize these intermediates to acetate, CO_2_, and H_2_, which serve as the substrates for methanogens—the strictly anaerobic archaea responsible for converting acetate, CO_2_, and H_2_ into CH_4_ and CO_2_ (“acetoclastic methanogenesis”) or CH_4_ and H_2_O (“hydrogenotrophic methanogenesis”).^164^ Because methanogens lack the enzymatic machinery to depolymerize lignin, crystalline cellulose, or other recalcitrant biopolymers, they rely entirely on upstream hydrolytic and fermentative organisms to generate the simple compounds required for methane formation. The extent to which anaerobic decay proceeds through this cascade is controlled by the fraction of the degradable organic carbon that is available for decomposition under anoxic conditions, a metric called DOC_f_. Structurally complex or thermally altered substrates—such as highly lignified biomass or biochar—exhibit intrinsically low DOC_f_ and therefore limited anaerobic biodegradability.

### 4.2 Abiotic Mechanisms

While geophysical and climatological conditions alone are not decay mechanisms, they do significantly inform the rate and mechanisms of decay, especially climate. While not the focus of this review, macroclimate informs local climate which in turn influences the biomass material climate, and these abiotic conditions are the foundation for water and energy availability for decomposer communities.^25,165^ In addition, there are primarily two decay mechanisms that are abiotic: photodegradation from ultraviolet (UV) radiation and combustion.

#### 4.2.1 Photodegradation

Photodegradation is the process by which biomass undergoes chemical and physical changes when exposed to sunlight or UV radiation over time. In photodegradation, UV light is absorbed by chromophores, which initiate photochemical reactions and the formation of free radicals.^166,167^ In turn, free radicals initiate oxidative degradation reactions in biomass by attacking the chemical bonds within lignin, and to a lesser extent, cellulose and hemicellulose.^167^ As lignin degrades, the biomass surface becomes more susceptible to mechanical damage and microbial decay, increasing the rate of mass loss.^26,67,168^ Exposure to UV radiation is dependent on site-specific conditions, but from a global perspective, locations at low latitudes and high altitudes with predominantly cloudless conditions and low column ozone are likely to receive more UV radiation.^169^

#### 4.2.2 Combustion

Fire is an increasingly important abiotic decay mechanism for biomass, particularly in recent contexts. For example, many forests across the western United States now experience higher fuel loads, drier conditions, and more frequent or severe wildfires compared to historical baselines. As a result, accumulated woody biomass—including standing dead trees, downed logs, and unharvested residues—is more vulnerable to combustion, leading to conversion of solid biomass into greenhouse gases (CO_2_, CH_4_), criteria air pollutants (CO, PM_2.5_, volatile organics), charcoal, and ash, with significant energy release. Unlike gradual abiotic weathering (e.g., photodegradation), fire is acute and results in near-total destruction or transformation of wood carbon in a very short period. In this context, fire represents not only a natural decay agent but also a significant, and potentially increasing, risk factor for carbon permanence for BiCRS and other nature-based solutions.

## 5 Carbon Product Fates: Decay Prevention Mechanisms

Given pathways of biotic and abiotic decay, several decay prevention mechanisms have been proposed in the literature or pursued in the CDR space. For each form of biomass described in Section 2, we assess suitability under each of four storage fates including **surface storage** (Section 5.1), **dry storage** (Section 5.2), **anoxic storage** (Section 5.3), and **geologic injection** (Section 5.4). Durable storage can be achieved through one or multiple decay prevention mechanisms operating in combination (Figure 6). For each combination of feedstock form and storage fate, we additionally highlight the carbon implications of failure occurring to the primary mechanism preventing decay (Figure 7).

**Figure 6:**
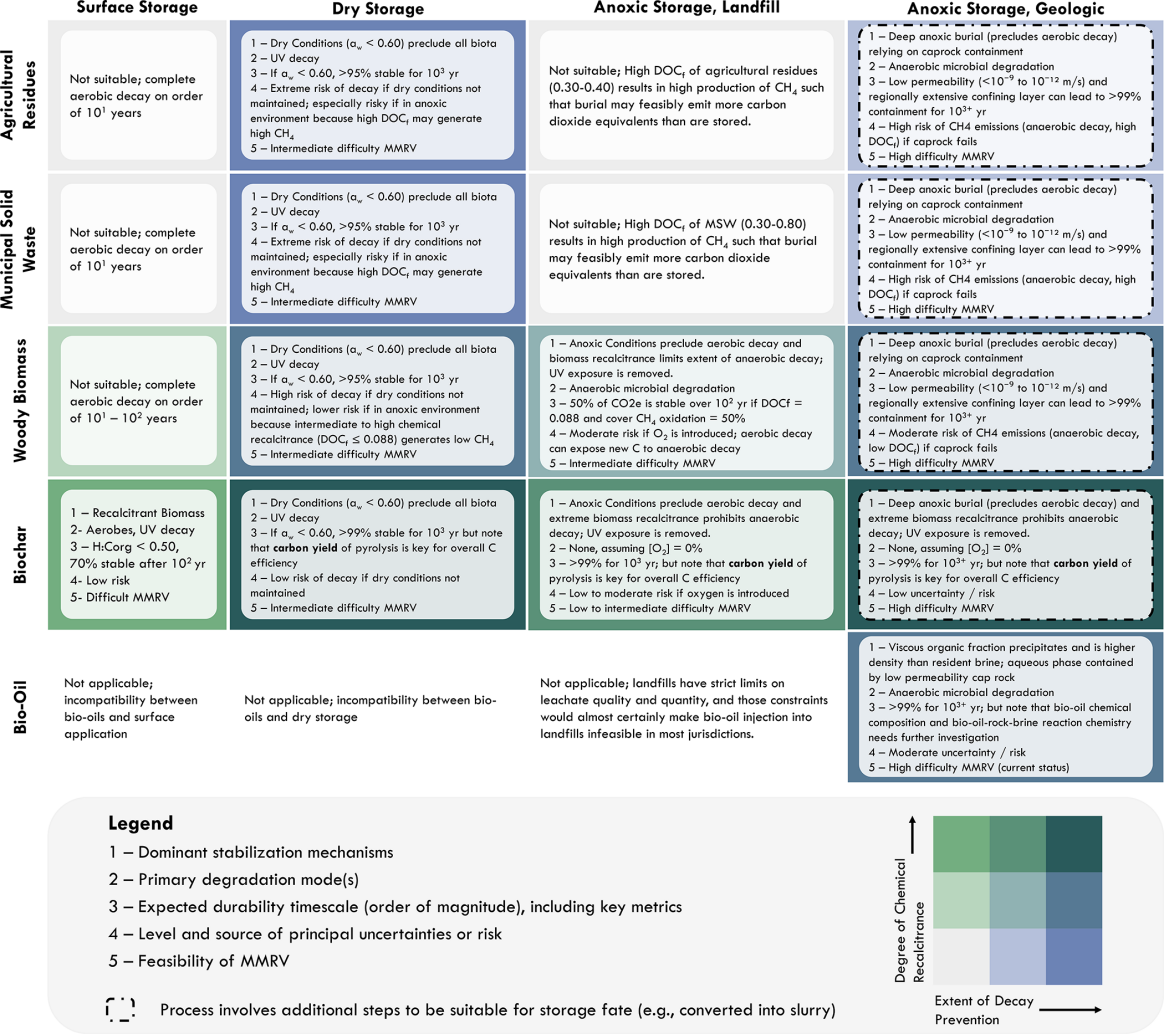
Durability matrix crossing feedstock forms (along left column) and storage fates (along top row). For each combination of feedstock form and storage fates, we describe (1) dominant stabilization mechanisms, (2) primary degradation mode(s), (3) expected durability time scale (order of magnitude), including key metrics, (4) level and source of principal uncertainties or risk, and (5) feasibility of current MMRV. Overall durability is shown on a bivariate color grid (gray to green showing increasing chemical recalcitrance and gray to blue showing increasing extent of decay prevention, with the deepest blue-green color indicating highest overall durability in both categories). Dotted lines highlight combinations requiring additional processing prior to storage (e.g., solid feedstock may require conversion into slurry prior to geologic injection). Infeasible combinations are simply depicted in white.

**Figure 7:**
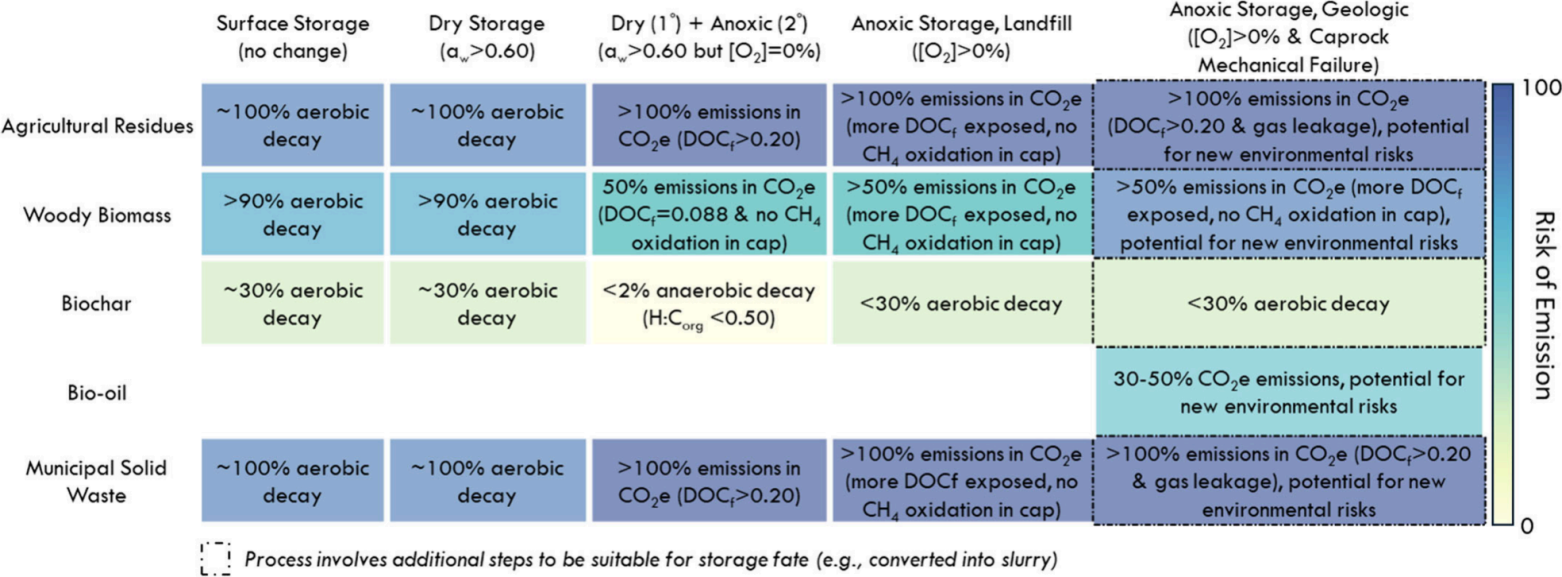
Carbon implications of breach to decay prevention mechanism for each combination of feedstock form and storage fate. Feedstock deployed in surface storage fate is already exposed to abiotic and aerobic decay mechanisms and is therefore unchanged from business as usual (unless major disturbance such as wildfire were to occur; not pictured). For feedstock deployed under dry storage fate, risks are dependent on whether conditions are oxic or anoxic. If water is introduced (*a*_*w*_ > 0.60) and conditions are oxic, carbon emissions will approximate surface storage (i.e., primarily aerobic decay). However, if water is introduced (*a*_*w*_ > 0.60) and conditions are anoxic, carbon emissions will approximate landfill storage. In this case, the feedstock form becomes critical, as less chemically recalcitrant biomass will be more vulnerable to anaerobic decay (i.e., higher DOCf) and higher CH_4_ emissions. For feedstock deployed under anoxic storage in landfill conditions, introduction of oxygen can enable aerobic decay, which can increase DOCf if anoxic conditions are re-established. Anoxic storage in geologic conditions are similar to landfills, except intrusion of oxygen and caprock mechanical failure may introduce new environmental risks that must be mitigated.

### 5.1 Surface Storage

Surface storage refers to the application of biomass-derived materials (e.g., biochar) to soils under ambient field conditions (i.e., oxic, near-surface environments) (Figure 6, left column). Under such conditions, all lignocellulosic biomass is subject to aerobic microbial decay, with rates strongly dependent on temperature, moisture, and substrate chemistry. Consequently, surface storage does not constitute a viable CDR pathway for agricultural residues, woody biomass, or municipal solid waste. Surface storage of bio-oil is also not applicable because it is chemically unstable under ambient conditions and, being uncontained, it can seep into soils, harm vegetation and microbial communities, and generate localized environmental contamination. In contrast, biochar can persist for centuries to millennia when applied to soils.^170^ Given the extensive coverage of biochar application to surface soils in prior reviews, we briefly describe stabilization mechanisms, primary degradation modes, durability metrics and expected time scale, risks, and feasibility of MMRV below for comparison to other nonenergy BiCRS storage approaches.

The dominant stabilization mechanism for biochar surface storage is feedstock chemical recalcitrance (see Section 3.1.1). The main risks to the long-term stability of biochar surface storage are aerobic decay and photodegradation. Specifically, in ambient conditions, labile or weakly condensed organic fractions on and near biochar surfaces (alkyl chains, oxygenated functionalities, residual tars) are susceptible to microbial attack and can be mineralized to CO_2_. Further, as with raw biomass, when biochar is exposed to sunlight, UV radiation can initiate photooxidation reactions, breaking chemical bonds in the biochar structure and leading to the formation of free radicals that initiate chain reactions with nearby molecules, causing further degradation.^171^ Environmental factors such as temperature, humidity, and oxygen availability influence the rate and extent of degradation.

As described in Section 3.3.1, the molar ratio of hydrogen to organic carbon is the primary method of measuring degree of chemical recalcitrance of biochar.^37^ A lower H:C_org_ reflects increased formation of aromatic structures, such as benzene rings and polycyclic aromatic hydrocarbons (PAHs), which have fewer hydrogen atoms, as well as greater structural complexity and chemical stability than more labile compounds, such as cellulose and hemicellulose. The amount of biochar carbon estimated to be stable after 100 years (BC+100) decreases linearly with increasing H:Corg. We therefore suggest that the key threshold is H:Corg <0.5 and assume that ∼ 20–30% of the biochar carbon will degrade over 100 years.^37^

Biochar surface storage presents relatively low risk (Figure 7), as it generally provides substantial cobenefits to soil communities. However, as it is likely to be deployed in an open system, difficulties around MMRV are large. For example, water-extractable fractions and small fragments can be mobilized and leached downward or laterally while erosion, wildfire, or incorporation into soil organic material can transfer carbon out of the original biochar pool. These processes can make directly tracking biochar fate extremely difficult.

Ultimately, biochar surface storage is a valid approach to nonenergy BiCRS. Beyond assessing durability, however, we recommend rigorous accounting of the initial carbon contained in the feedstock, its counterfactual fate, and the carbon yield of the conversion process. Given that other approaches start with a carbon yield approaching 100%, it is important to quantify the trade-offs between increased stand-alone durability and immediate loss of carbon.

### 5.2 Dry Storage

For all feedstocks described herein, biotic decay can be prevented if the storage conditions are maintained below a threshold water activity (*a*_*w*_), a process called Dry Storage (Figure 6, second column from left). The concept of *a*_*w*_ as a stabilization method is based upon empirical evidence that biota cannot survive when the available water is insufficient to support their metabolic processes and cellular functions.^172,173^ This theory emerged from studies comparing solute tolerance across life domains.^174,175^ Early work suggested a stark divide, as prokaryotes like halophilic Archaea and Bacteria were thought incapable of growth below 0.755 *a*_*w*_ (saturated NaCl^176^), while eukaryotes such as xerophilic fungi could germinate at 0.650–0.605 *a*_*w*_ in high-sugar environments.^177^ However, experimental re-evaluations revealed that extreme halophiles, including *Halobacterium spp*. and *Halococcus spp.*, proliferate at far lower *a*_*w*_ values (0.687–0.635) in hypersaline media, with extrapolated theoretical minima *a*_*w*_ low as 0.611.^177^ These findings challenged the notion of eukaryotic superiority in low-*a*_*w*_ environments, suggesting a universal physicochemical limit tied to cellular hydration requirements rather than domain-specific biology. We therefore suggest that the key metric controlling dry storage is maintaining a_w_ < 0.60. Once the biomass is dried and storage implemented, the *a*_*w*_ must be maintained below the theoretical minima in perpetuity, or decay can restart.

Establishing the threshold water activity can be achieved for raw and converted biomass by various drying processes. In unmodified soil environments, pore space *a*_*w*_ generally does not decrease below 0.95, even in arid environments with well-adapted vegetation.^178^ Therefore, if underground storage is desired, additional engineering is required to maintain dry conditions inhospitable to decay. One approach for the maintenance of dry conditions is to physically encase the dry biomass in plastic or polymer-based materials (e.g., high-density polyethylene, HDPE). Dry storage can alternatively be established and maintained via salt addition.^16^ Salts reduce the *a*_*w*_ of a contained space by binding water molecules, creating osmotic pressure, and lowering the vapor pressure, thereby reducing the amount of free water available for biological and chemical processes (^16^ and citations within). Salts like NaCl and MgCl_2_ achieve low *a*_*w*_ through ionic dissolution and solute-specific interactions. NaCl, a kosmotropic agent, stabilizes macromolecules but has limited solubility (saturating at ∼ 5.2 M, *a*_*w*_ 0.755), whereas MgCl_2_’s higher solubility enables *a*_*w*_ reduction to 0.328 in saturated solutions. The interplay between solute type and cellular adaptation mechanisms remains poorly resolved in the literature and warrants further study.

Both the risks associated with dry storage and difficulty of MMRV are primarily dependent on the engineering employed (Figure 7). Physical encasement can maintain dry conditions and contained encasements can easily be outfitted with sensors for relative humidity or product gases of decay. However, long-term performance of the polymer will be key for establishing and maintaining dry conditions. While the permeation, absorption, and diffusion of gas and solutes across polymers is well-studied, it is less understood how weathering of material may progress in the context of burial or UV exposure over centuries (but see^179^). In addition, termites and other insects can penetrate a variety of plastics or other wrapping material and their activity could compromise the integrity of a physical barrier.^180,181^ Similarly, salts can generate and maintain dry conditions, but they introduce significant risks in the event of a water breach, including the potential production of toxic gas^182^ or leachate^183,184^ —suggesting that a physical barrier may be necessary under most dry storage approaches. The long-term performance of the barrier should be a primary research priority for any encasement approach, especially if the storage conditions lack oxygen as anaerobic microbes, such as methanogens, can more rapidly and completely degrade labile biomass into CH_4_ (Figure 7, columns 2 and 3).

### 5.3 Anoxic Storage, Landfill

#### 5.3.1 Anoxic Storage of Raw Biomass

Another approach for raw biomass storage includes burial in anoxic environments, similar to methods for landfilling waste (Figure 6, third column from left). Key components of this system include an excavated, anoxic vault area filled with biomass (hereafter “vault”) and a multilayer, low-permeability cover system designed to isolate stored carbon materials from surface processes and the atmosphere (hereafter “cap”). In anoxic vault environments, anaerobic decay pathways will occur. While anaerobic decay pathways are slower and less energetically favorable than aerobic decay, they can lead to the production of highly potent greenhouse gases, such as CH_4_.^185^ Therefore, these pathways are not suitable for all forms of raw biomass (e.g., MSW and agricultural residues, Figure 6). The durability of anoxic storage is primarily determined by biomass chemical recalcitrance, which controls the fraction of the biomass carbon that is vulnerable to decay.

Biochemical methane potential (BMP) tests^186^ (e.g., ASTM E2170-01) are used to determine the anaerobic degradability of biomass, and ultimately, the fraction of the DOC that is available for decomposition under anoxic conditions, or DOC_f_. Lignin is especially recalcitrant and restricts the degradation of cellulose and hemicellulose by limiting microbial access in anoxic environments^85^ but see^187^). Softwood tree species, which make up the majority of forests in the Western United States, are particularly lignin-rich, with reported DOC_f_ values ranging from 0.001 to 0.08.^188,189^ This means that between 0.10% and 8.0% of the DOC contained within softwoods is vulnerable to anaerobic decay. Meanwhile, other forms of biomass, including hardwoods, grasses, paper products and food waste have higher DOC_f_ values,^189–192^ implying higher projected CH_4_ production under anoxic conditions (Figure 8A).

**Figure 8:**
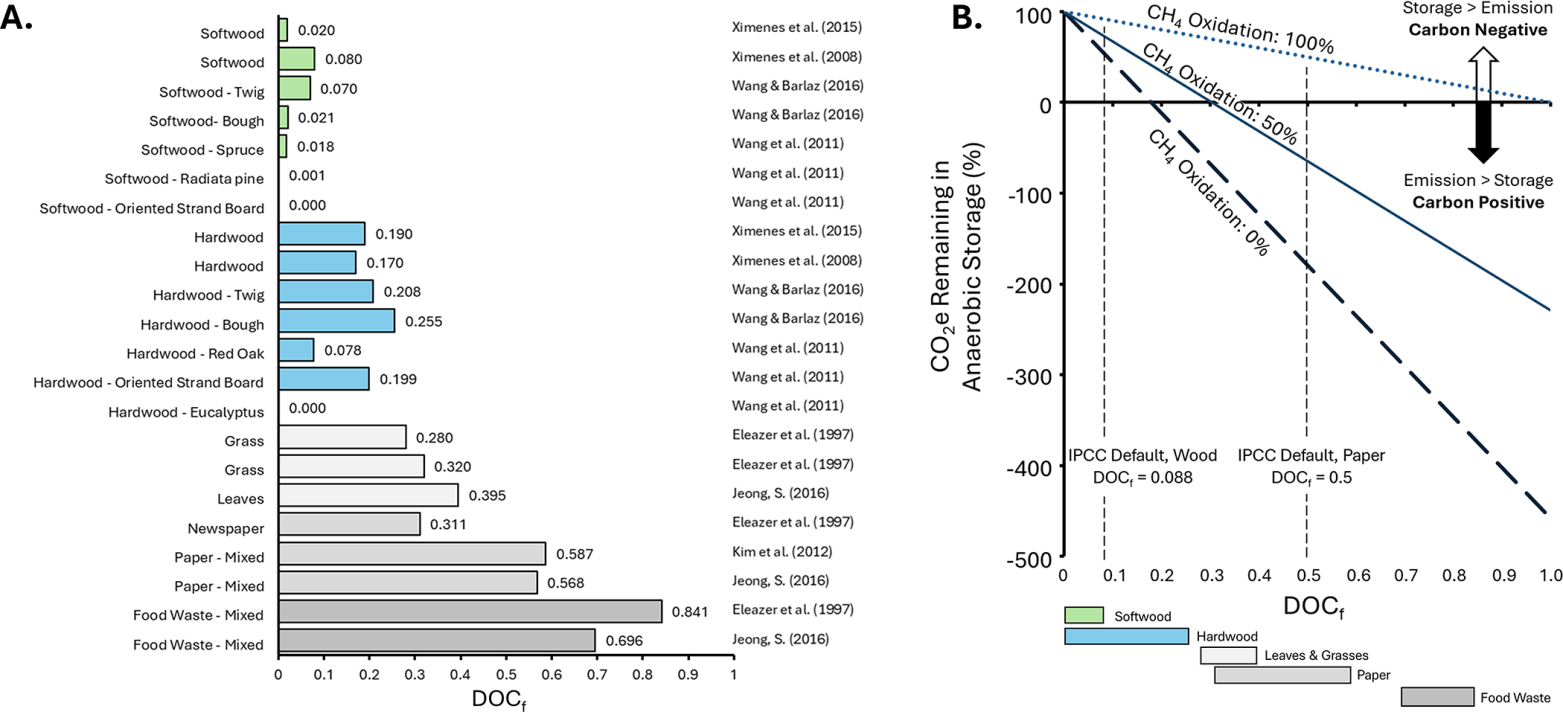
Biomass DOC_f_ and Implications for Carbon Storage. (A) The fraction of Degradable Organic Carbon vulnerable to decay in anoxic conditions (DOC_f_) is highly variable across biomass compositions, including softwoods (green) and hardwoods (blue), grasses and leaves (light gray), paper (intermediate gray) and food wastes (dark gray). As anaerobic decay proceeds, equimolar contributions of CH_4_ and CO_2_ will be produced. (B) Biomass DOC_f_ and CH_4_ oxidation rate in soils control the amount of the initial biomass CO_2_e remaining in storage. Positive values represent a carbon negative process and negative values represent a carbon positive process (driven by the high GWP of CH_4_).

Anoxic storage of raw biomass approaches are therefore only suitable for more chemically recalcitrant feedstock, such as biochar or raw biomass with a DOC_f_ less than 0.088. The IPCC derives this average DOC_f_ value of 0.088 for less decomposable wastes, primarily woody biomass.^193^ At a DOC_f_ of 0.088, in the worst-case scenario, if 100% of the DOC_f_ degraded and no CH_4_ oxidation occurred in the cap, then the emission of the entire DOC_f_ would represent ∼ 50% of the total CO_2_e in the biomass (Figure 8B). If DOC_f_ exceeds 0.088, anoxic storage should not be pursued owing to the low durability over 100 years, unless (1) the conditions of the vault are tightly controlled to minimize the rate of decay; or (2) the cap is engineered to contain product gases or promotes consistently high rates of methanotrophy.

Any raw biomass contained in anoxic conditions will undergo anaerobic decay, a microbially mediated process, dependent on the local oxidation–reduction (redox) chemistry, that requires coordination among several trophic groups of microbes^164^ (see Section 4.1). The rate of anaerobic decay can be inhibited or expedited by conditions within the vault including: (1) moisture, (2) temperature, (3) pH, and (4) nutrient availability.^164,194,195^ If vault conditions are dry, cold, nutrient poor, and/or acidic, it becomes more feasible to safely store less recalcitrant forms of biomass because decay will proceed slowly if conditions are maintained. Importantly, the slower the CH_4_ flux, the greater the fraction that will be oxidized in the cap.^196^ Additional research is required to better constrain the relative importance of each of these factors and derive predictive relationships among these factors and expected rates of decay.

Emission of CH_4_ and CO_2_ to the atmosphere is controlled by properties of the cap, as it will dictate the inflows and outflows of liquids and gases. First, the infiltration of water is an important determinant of the rate of decomposition, as it is a required solvent for hydrolysis and a medium for transport of molecules and microbial activity.^197,198^ Conventional landfill covers minimize percolation of water into the chamber by having a cover conductivity of ≤ 1 × 10^–7^ cm s^–1^ (RCRA Title 40, Section 258) and set thickness requirements. Alternative landfill cover designs minimize percolation by providing sufficient water storage capacity in the soil cover given the local balance of precipitation and evapotranspiration.^199^

As anaerobic decay proceeds, CO_2_, CH_4_, and VOCs will be produced, and will migrate out of the vault as the pressures and concentrations increase in the vault relative to outside. In addition to physical barriers to emission, biological barriers can be effective. Soil methanotrophic bacteria can oxidize up to 100% of the CH_4_ produced into CO_2_ and microbial biomass,^200–202^ but their effectiveness is variable with oxygen and moisture availability, temperature, and CH_4_ concentrations.^203,204^ While methanotrophic bacteria can be prolific in simply revegetated covers,^205^ more engineered biological barriers can be optimized relative to local climatic conditions.^206^ The design, implementation, and deployment of cost-effective biocovers is a promising frontier of future research.

Even with the most recalcitrant forms of raw biomass, durability under anoxic storage conditions ultimately depends on the continuous exclusion of oxygen, as the DOC_f_ is dependent on the maintenance of anoxic conditions (see Figure 7 for carbon implications of oxygen intrusion). Several types of breaches can occur. With earthen covers, freeze–thaw cycling can result in macropores or preferential pathways for gas movement.^199^ Other mechanisms leading to cracks include drying and shrinkage, inadequate or overcompaction, and differential settlement.^207^ Further, subterranean termites are highly efficient at locating and exploiting wood resources,^208^ even when they are buried deep below the ground surface.^209^ Modern landfill engineering practices, such as the use of composite liners (geomembranes and compacted clay liners), gas extraction systems, and surface drainage control, have evolved to minimize these reversal risks.^210^

#### 5.3.2 Anoxic Storage of Biochar

The primary requirements for effective anoxic storage of biochar are (1) reduced oxygen potential, (2) removal of UV exposure, and (3) prevention of mechanical agitation (e.g., tilling, mixing, etc.). Burial of biochar in vaults can achieve all three of these conditions, depending on soil type and cap engineering, microbial and fungal activity, and climate (Figure 6). To our knowledge, there are no known or documented cases where anaerobic microbes or fungi decompose biochar. The general inability of microbes and fungi to anaerobically decompose noncarbohydrate macromolecules helps explain the stability of coal underground for millions of years; even relatively “low-grades” of coal, such as lignite with R_0_ < 0.4%, have remained relatively stable.^211–213^

The most notable risk of this storage approach is the reintroduction of oxygen into storage environments. The presence of oxygen may activate aerobic microbial and fungal decomposition pathways (Figure 7). Water percolation into the storage chamber does not represent a direct risk to biochar durability, as there is no evidence that water activity increases the decomposition risk of biochar for anaerobic microbes and fungi (assuming carbohydrates are absent), since C–C bonds are not susceptible to hydrolysis. Aqueous landfill-simulations of wood decomposition demonstrate the stability of C–C and C–O bonds in lignin.^214^ However, indirect effects of water percolation into the burial chamber include the transport of oxygen into the burial chamber or the transport of biochar to the surface. Landfills and other storage environments attempt to minimize this risk by promoting microbial active soil layers that deplete oxygen in the first ∼ 30–50 cm of cover.^199,215^

Empirical evidence suggests that thermochemically converted biochar will be more durable under a wider range of conditions than raw biomass, including that with low DOC_f_. However, as with any conversion product, there will be a carbon accounting trade-off between emissions generated to convert raw biomass into biochar (higher near-term emissions; anoxic storage of biochar) and potential emissions resulting from higher rates and mass of biomass decay over time (higher long-term emissions; anoxic storage of raw biomass).

For anoxic storage of both raw biomass and biochar, MMRV considerations are similar. In both scenarios, measurement infrastructure can be deployed within the vault and at the interface between the cap and the atmosphere. Within the vault, measurement of oxygen, temperature, and product gases is feasible, with precedent set within the landfill literature (e.g.,^216^). At the surface of the cap, gas flux measurements (e.g., static chambers) or eddy covariance measurements across landscapes can highlight emission of any product gases in comparison to control areas (e.g.,^217^). Given the contained nature of these approaches, we characterize the MMRV as relatively low to moderate difficulty.

### 5.4 Anoxic Storage, Geologic Injection

#### 5.4.1 Biomass Slurry Injection

An emerging form of storage for raw biomass is the underground injection of sawdust and other biomass waste-based slurries, sometimes referred to as Solid Injection to Raise Ground Elevation (SIRGE).^24^ This technique is based on processes used for environmental hydraulic fracturing, wherein pressurized slurries of material are injected into the subsurface. The injection process propagates horizontal fractures or expands existing pore networks, forming laterally extensive, anoxic reaction zones in which microbial activity is limited by oxygen exclusion, low permeability, and in some cases, high salinity.^218–220^ Serious uncertainties remain regarding the long-term fate of organics under subsurface conditions, potential fracture migration or connectivity to groundwater resources, and the mechanical integrity of the injection zone under repeated cycling. Further study is needed to characterize biogeochemical feedbacks, optimize slurry formulation, and establish robust monitoring frameworks capable of verifying storage durability. Given the theoretical feasibility of these approaches, we include their consideration in Figures 6 and 7 but highlight that additional processing steps (dotted lines) and potential for environmental risks must be considered. Considerations for bio-oil injection will likely apply similarly to biomass slurry injection and can be found in Section 5.4.2 (below).

#### 5.4.2 Bio-oil Injection

Subsurface storage of bio-oil provides ample storage capacity across a wide range of geographies.^100,101^ Bio-oil can be emplaced into several types of subsurface features including reservoirs and salt caverns. Each storage type presents unique requirements. Salt caverns require the bio-oil to be saturated with salt to prevent dissolution of the cavern’s halite walls.^22^ Reservoirs require low solids loading, which can be achieved by either filtering in the pyrolyzer before bio-oil condensation or by directly filtering bio-oil. The specific filter size requirement is determined by the specific reservoir properties.^22^ Bio-oil may also be pH-adjusted to meet regulatory requirements.

Bio-oil durability and interactions in geologic reservoirs will need to consider variables and features including aquifers (deep/saline and shallow/fresh; flow fields and compositions), microbial communities, temperature and pressure, stability (induced seismicity, subsidence), and flow systems of produced or circulated fluids (hydrocarbons, brines, solution mining, geothermal, etc.). Some questions about the injectability and postinjection mobility and durability of bio-oil may benefit from understanding the behavior of petroleum in the subsurface, including quantitative framing of controls on buoyancy, phase separation,^150,151,221^ and migration including wettability and relative permeability.^222^

Lack of biotic and abiotic degradation in some petroleum or bitumen deposits provide supporting analogies for subsurface durability, but compositions and many properties of bio-oil and its expected subsurface behavior are different from those of fossil hydrocarbons, including density contrasts with reservoir fluids,^119^ biodegradation potential,^148,223,224^ and mineralogical and geochemical consequences of reactions with host rocks.^225^ Modeling, measurement, and monitoring efforts should consider what the fate of bio-oil and its derivatives and reaction products would be in flow paths lacking impermeable barriers.

The density contrast between bio-oil and resident brine may be a primary factor for long-term stability. Almost all liquid hydrocarbons, except typical bio-oils, are less dense than water or brine in subsurface pore spaces and fractures. This makes hydrocarbons prone to upward migration (as well as responding to larger length-scale pressure gradients) and trapping beneath impermeable layers with dome-like or fault-bounded geometries. In contrast, bio-oil can be denser than typical brine (i.e., 1.2 g/cm^3^ vs 1.0 g/cm^3^) and may therefore be less prone to upward migration. It may also be more likely to be “inversely” trapped by impermeable barriers like synclines or fault-bounded layers underlain by impermeable units. The generalization about bio-oil’s density compared with resident fluids is complicated by the fact that bio-oil can vary in density depending on the feedstock and reaction conditions^226,227^ and brine density can vary as well.^228^

Variations in bio-oil chemical composition also impart differences in physical properties including density and viscosity which may have implications for migration potential of injected bio-oil. It should also be considered that these distinct properties and their implications for mixing and migration potential may change under different temperature, pressure, and other reservoir conditions. Importantly, many bio-oils phase separate or dissociate into water-insoluble and water-soluble liquid phases over short time periods under variable storage conditions (known as aging; although we note that “aging” can occur instantaneously upon contact with water, brine, or rocks).^229,230^ It is possible that reaction-induced phase separation in geologic reservoirs occurs, which may produce water-soluble liquid phases similar in composition to the aqueous phase generated from biomass pyrolysis processes. For example, when pyrolysis oil is introduced to brine or aqueous media, significant fractions (up to 60–90%) of the carbon may dissolve in the aqueous fraction while the solid or viscous organic fraction precipitates (known thereafter as pyrolytic lignin).^231,232^ The highly viscous or solid organic phase of bio-oil could then be durably stored in pore space. The production of viscous or solid organic phases from bio-oil aging and water mixture processes is likely to occur during geologic storage as these processes are well documented at standard conditions, as well as via accelerated aging and heating processes (see Figure 9).^231,232^ Preliminary experiments show that these phases strongly partition elements released from reaction with simulated host rocks and brines.^225^ One potential durability implication of the reaction-induced dissociation of bio-oil in aqueous and organic phases is that the less-dense, less-viscous, and water-miscible reaction-induced aqueous phase may be mobile in the subsurface, while the organic fraction would be significantly less mobile. Research, measurement, monitoring, and modeling efforts should consider these potential occurrences based on their relevance to leakage and durability of bio-oils in geologic reservoirs.

**Figure 9:**
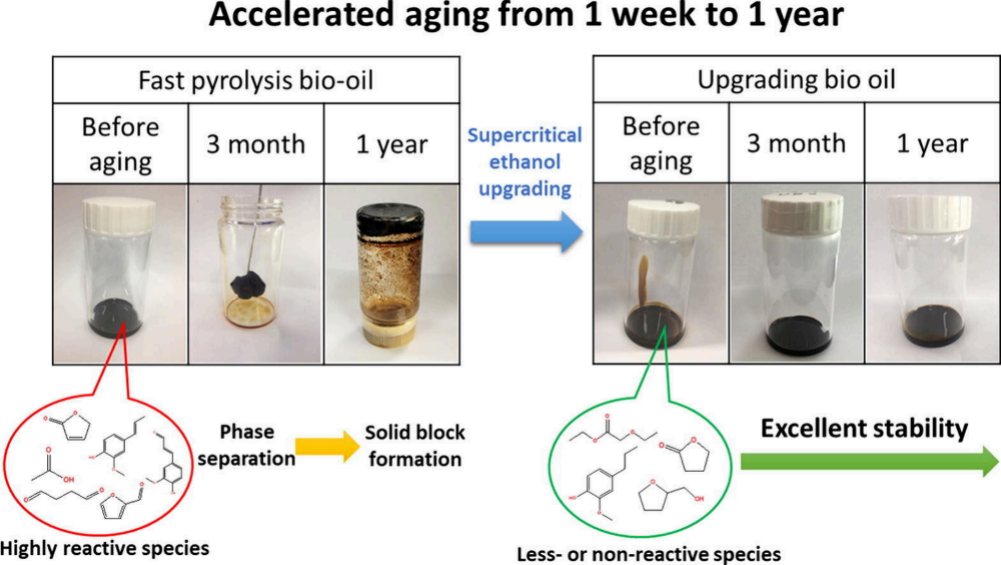
Directly reproduced from (Jo et al., 2018).^229^ Copyright 2018, Elsevier. Aging studies have shown biomass pyrolysis oil forms viscous liquids or solids, but it may also be upgraded or otherwise changed to produce emulsions or other fluids.

Bio-oil-rock-brine reaction chemistry should be investigated to provide considerations for potential reservoirs for bio-oil injection. For example, evaluating the dissolution of calcite by bio-oil and the resulting impacts on porosity in carbonates and calcite-cemented sandstones in the injection reservoirs may help drive decision making in bio-oil injection reservoirs and also inform durability and leakage. The rock units selected for potential injection should be characterized for their lithology and composition (including mineral content, structure, and resident brine). However, few studies have been conducted to date to understand bio-oil-rock-brine reaction chemistry.

Potential biological decomposition and degradation also needs to be considered for bio-oil geologic storage, though this has not been investigated under relevant storage scenarios to date. Basaglia et al. (2021)^224^ identified numerous fungi and bacteria that were not only able to tolerate existing conditions and at various dilutions in representative bio-oil aqueous phase but could also metabolize the carbon species present. While microbial metabolism and conversion of bio-oil components has been suggested and observed,^233–236^ pyrolysis oil at low concentrations has also been shown to have inhibitory or toxic effects on many microbial organisms, particularly due to the presence of furfural, 2-cyclopenten-1-one, and phenolic compounds. Durability and leakage considerations need to account for the potential presence of microbes that are able to tolerate the conditions of the storage (including pressure, temperature, pH, presence of minerals, etc.) as there are organisms known to occur in deep subsurface environments.^237^

The long-term fate of bio-oil upon aging under representative reservoir conditions including temperature and pressure also needs to be investigated, modeled, and monitored to ensure durability. The potential decomposition of bio-oil to gases as well as the dilution and mobility of the organic and aqueous phases could be impacted by these variables and are known to result in changes to pyrolysis oil properties including viscosity and chemical functional group properties,^231^ flow behavior and molecular weight distribution,^238^ among other properties.^99,239,240^

MMRV for bio-oil geologic injection must extend beyond initial carbon quantification to include monitoring of containment integrity, compositional evolution (e.g., changes in O:C ratio, viscosity), and potential gas generation or leakage pathways over time. Importantly, MMRV frameworks for bio-oil injection can evolve by leveraging established practices from related subsurface industries, including carbon capture and storage, underground hydrocarbon storage, and hazardous waste injection, where long-term pressure monitoring, tracer studies, geochemical sampling, and well integrity assessments are routinely applied (see Section 6.3). As empirical data from pilot and early stage deployments accumulate, MMRV requirements can be iteratively refined to better link observable indicators to degradation rates and leakage risks, enabling risk-proportionate monitoring strategies and progressively more robust durability claims.

## 6 MMRV Methodologies

All BiCRS projects must be accompanied by rigorous validation of durability claims and to ensure appropriate carbon accounting. MMRV of feedstock chemical recalcitrance and establishment and maintenance of the decay prevention mechanism need to be performed using validated methodologies to ensure permanent storage and detect any reversal mechanisms taking place. In prior sections we described the key metrics that must be met (Figure 2); in the following sections we describe the existing analytical methods that can be used to quantify variables that may impact the durability of BiCRS projects for raw (Section 6.1) or conversion products (Section 6.2); or may be key variables to monitor under different storage fates (Section 6.3). While directly observable data would provide the most certainty for durability, there are some scenarios where databases, models and assumptions may be used to support removal and durability claims when project-specific data is not available. There is currently a shortage of long-term experimental evidence showing the durability of biomass and bioproducts as it relates to chemical and physical properties under certain storage scenarios. Additionally, more research is still needed for the utilization of monitoring technologies to understand long-term storage processes and reversal mechanisms.

### 6.1 Raw Biomass Analysis Methodologies

There are extensive studies, standards, reviews, and books covering analytical techniques used for characterization of biomass, particularly as it relates to chemical recalcitrance, decomposition and deconstruction for utilization as chemicals and energy.^241–244^ Biomass elemental analysis, particularly carbon, hydrogen, and nitrogen (CHN) content, is typically achieved using commercially available CHN analyzers with associated standard methodologies (e.g., LECO). Lignin content, structure, linkages, and composition in biomass can be determined via wet chemical techniques and instrumental analyses including acid hydrolysis,^245^ acetyl-bromide,^246^ analytical pyrolysis,^247^ thioacidolysis,^248^ nuclear magnetic resonance (NMR),^249,250^ and many other methods.

Cell wall carbohydrates can be analyzed by acid hydrolysis to determine the relative abundance of sugar monomers present in cellulose and hemicelluloses.^244,248^ Total cellulose and hemicellulose content in woody biomass has traditionally been determined via many wet chemical methods,^251^ though many other techniques are used lately to characterize cellulose and hemicellulose composition, crystallinity, structure, etc.^252,253^ NMR is also used to analyze many other biomass cell wall components, linkages, structure and can provide key insights into changes in organic compounds to determine recalcitrance or durability.^241,254,255^

Analysis of labile, extractable (which may represent less durable) components including sugars, metabolites, and other small molecules can be achieved via solvent extractions following chromatographic analyses.^256–258^ Inductively coupled plasma optical emission spectroscopy (ICP-OES) and other techniques such as laser-induced breakdown spectroscopy (LIBS) could be used to determine the abundance of inorganic elements present in biomass.^259,260^ These methods and others will be essential to utilize to make scientific insights relating biomass properties to durability and to confirm properties of biomass required for durable BiCRS projects.

In addition to assessing chemical characteristics of the raw biomass, there are complementary approaches designed to quantify durability of biomass under certain conditions. For example, biochemical methane potential (BMP) tests measure the maximum methane (CH_4_) produced by a given organic substrate during anaerobic digestion. Historically, BMP was used by biogas facilities, as a way to predict and optimize methane generation for energy uses. BMP experiments are performed in batch under controlled conditions.^261^ The substrate is mixed with excess inoculum—an anaerobic bacteria culture, often sourced from an active digester—and left to incubate. Incubation times range from one month to over 100 days, during which methane production is tracked.^262^ The total amount of carbon released is compared with control samples to determine the potential degradation potential of the sample (e.g., ASTM E2170-0, 2008).^186^ BMP is linked to degradability extent, first order rate coefficients for decay, and is used to calculate the fraction of the degradable organic carbon that is vulnerable to anaerobic decay^193^ (see Section 5.2).

### 6.2 Conversion Product Analysis Methodologies

Many analytical techniques used to analyze biochar and bio-oils are based on their analogous fossil fuel materials, particularly coal and petroleum, respectively (e.g.,^32,263^ ). There are many analytical methods developed for the analysis of coal to assess chemical, physical, thermal, metallurgical, petrographic and other properties particularly as it relates to use as a fuel and carbon source.^264–267^ Many of these methods may be used to characterize biochar properties as well. The analysis of CHN and S content in biochar may be carried out using similar methods and instrumentation as coal (i.e., ASTM D5373). CHN content is typically determined using an instrument that combusts the sample to convert carbon, hydrogen and nitrogen in the solid sample to their respective gases of CO_2_, water and nitrogen which are analyzed by a detector such as a thermochemical conductivity detector (TCD).

However, careful attention to differences between biochar and coal may need to be considered during execution of analysis methods which may require changes in instrumentation parameters, optimization, inclusion of other methods, etc. For example, biochar may have higher oxygen content than most coals due to residual sugars and lignin, which should be considered during CHN­(O) analyses that typically depend on calculating oxygen simply by subtraction when applied to coal (e.g., ASTM D5373; LECO Reference Number: 203-821-364). For biochar, other analyses may be needed to support the presence of oxygen including extraction (via solvent, heat, etc.) and analysis of oxygenated hydrocarbons (via detection by mass spectrometry, IR or other detection methods). In particular, it may be necessary to analyze biochar for labile, extractable and potentially less durable oxygenated hydrocarbons such as remaining sugars that could undergo decomposition and reversal. Analysis of sugars present in biochar can be performed similarly to the processes used to analyze sugars in biomass: extraction in conjunction with acid hydrolysis followed by HPLC analysis of extracts and hydrolysates.^245^

Random reflectance, R_o_, as well as fluorescence of biochar are also durability metrics previously applied to coals and other macerals to assess alteration, weathering or degradation^268^ (e.g., ASTM D7708-14). To measure R_o_, samples may be prepared as epoxy pellets and a microscope with light detection system is used to analyze the samples via white light reflectance spectrometry, which measures the dispersion of light reflectance from the samples from about 430–670 nm. Fluorescence spectrometry may also be used to analyze the λ_max_ and ratio of red to green (R/G) wavelengths in reflectance curves of a sample of biochar to indicate the degree of aromatization or condensation of carbon, where higher R/G and lower fluorescence are characteristics of inertinite macerals.^268^ Many measurements (on the order of tens-hundreds) of fluorescence or R_o_ are made to obtain an average and distribution of values across a representative sample (Figure 10). As shown in Figure 10, microscopy measurements can be used to identify different maceral types with spatial resolution across samples to produce histograms and perform statistical analyses for calculation of biochar R_o_ values.

**Figure 10:**
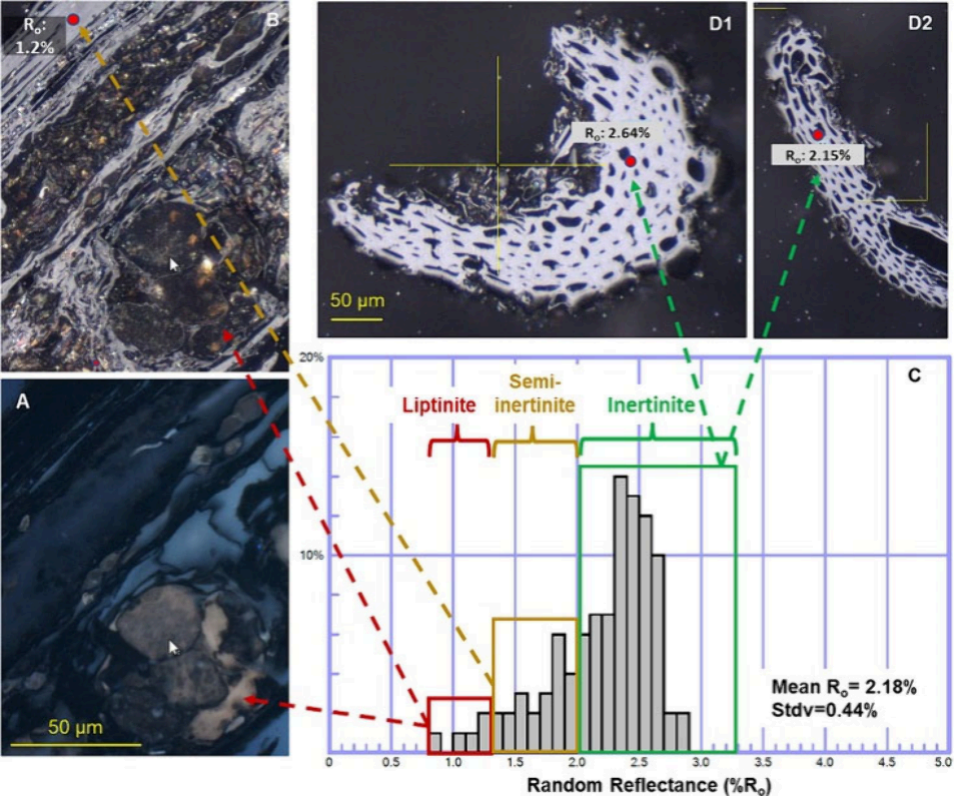
Directly reproduced from (Sanei et al., 2024).^32^ Copyright 2024, Elsevier. This figure identifies liptinite (red), semi-inertinite (dark yellow), and inertinite (green) organic pools in a biochar sample using a combination of the fluorescence and white incident light microscopy. The R_o_ histogram (shown in C) displays the correlation between organic pool type and reflectance range.

Gas physisorption techniques can be used to analyze the surface area (m^2^/g) and porosity properties (including pore size, volume, percentage) of biochar^269^ which may vary depending on the analytical techniques and parameters, as well as the process and feedstocks used to generate the biochar.^270–272^ Brunauer–Emmett–Teller (BET) analysis uses nitrogen or CO_2_ physisorption to measure the surface area and infer micro- and macropore volumes. Biochar surface area can vary substantially ranging from <1m^2^/g to hundreds of m^2^/g while micro, meso and macro pore volumes are typically totaled to less than 5 cm^3^/g.^273^ Mercury porosimetry and scanning electron microscopy can also be used to analyze biochar pore properties.^272,273^

Biochar density and particle size distribution are other physical properties that may be measured as they impact CDR pathways using biochar (i.e., density could impact efficiency of transportation of biochar, particle size may be a safety consideration, etc.). Biochar bulk density is typically on the order of 0.1–0.6 g/cm^3^ ^272^ and can be determined using a variety of standard methods for analyzing mass per unit volume (e.g., ASTM D422).^269^ Particle or skeletal density of biochar are typically higher, on the order of 1–2 g/cm^3^, and are determined by helium pycnometry.^273^ While it is not known if denser biochar would have higher durability under geological storage scenarios, it may be more efficient to transport and store biochar with higher densities than that with lower density. These properties may be particularly important for optimization of economic and process efficiencies, and in environmental and agricultural applications as they relate to gas and pollutant adsorption and other remediative properties, though it is not clear if they impact biochar durability directly.^39,274,275^

There have been many reviews covering the analytical approaches used to characterize bio-oils.^276–278^ Analysis of CHN content in pyrolysis oils derived from biomass is also of primary importance for utilization of bio-oils in CDR pathways. Bio-oil C content is typically on the order of 50–60 wt %, H is approximately 6%, O is typically about 40 wt %, with trace amounts of N depending on the feedstock.^93,98^ Similar combustion-based instrumentation as that used for CHN content determination of other materials may be used for pyrolysis oil CHN analysis.^279^

As discussed previously, it may be beneficial to measure other properties to understand the durability and mechanisms of bio-oil aging during geologic storage and for environmental purposes. In particular, bio-oil density, viscosity, pH or total acid number, molecular weight distribution, low molecular weight hydrocarbon components, water content, and proximate analyses may be related to bio-oil durability and could serve as important foci in research related to bio-oil injection in geologic reserves. Bio-oil density can be measured using density meters similar to those used for other oils (e.g., ASTM D4052) and is typically on the order of 1.1 g/cm^3^.^93,98,280^ Bio-oil viscosity analysis should also be considered based on its potential for changes when mixed with water and as the result of aging processes; but viscosity analyses are also associated with challenges and should be interpreted carefully.^231,280^ Analysis of carbonyl content in bio-oil by titration methods and/or by NMR may be used otherwise to understand bio-oil aging processes.^231,281,282^

^13^C and ^31^P NMR analysis of bio-oil also provides insights related to other functional groups, particularly those containing oxygen, that could change or be measured to understand bio-oil interactions with rock during storage.^276,282,283^ High performance liquid chromatography (HPLC) and gas chromatography with mass spectrometry (GC/MS) detection can be used to identify and quantify various specific components in bio-oils and aqueous products including sugar-derived species, phenolics derived from lignin and other compounds.^284,285^ Molecular weight distribution (MWD) and other molecular weight metrics of pyrolysis oils can also be determined using HPLC and NMR methods to understand the size of species in bio-oils, particularly the presence of higher molecular weight, recalcitrant, durable species (i.e., >400 Da) and provide insights on aging.^257,286,287^ Determination of water content in pyrolysis oils using titration methods can be helpful to understand how the presence of water in bio-oil influences other properties such as viscosity, density, interactions with rock and brine during geologic storage.^279^

### 6.3 Storage Fate Analysis Methodologies

Measurement of conditions within storage fates is critical, especially in scenarios where less chemically recalcitrant biomass is stored. Key methodologies vary across approaches, as the decay prevention mechanism is different for each approach. For Dry Storage, for example, continuous measurement of relative humidity, as an indirect measure of *a*_*w*_, within a statistically significant number of representative storage chambers is key. Supplemental indirect measures, such as temperature (which is required to calculate relative humidity), can support detection of failures. Continuous or regular measurement of products of decay, CO_2_ and CH_4_, is another priority for pilot deployments. Sampling design for product gases should be informed by storage design, i.e., multiple small chambers will require a different approach than one large chamber engineered to direct gas buildup into a headspace.

For Anoxic Storage in Shallow Landfill designs, continuous measurement of oxygen in the vault is essential. Quantification of C:N and DOC_f_ of a representative sample is essential to understand the total amount of biomass carbon that is vulnerable to decay, while optional measurement of temperature, relative humidity, and pH within the vault can constrain expected rate of decay and may provide early warning signs of failure. For closed systems (e.g., enclosure with impermeable geomembrane), product gases can be periodically sampled from a centralized collection point. Within more open systems (e.g., monolithic evapotranspirative covers that minimize percolation but not completely enclosed in an impermeable barrier), exact quantification of product gases is more difficult to constrain. Replicated surficial gas flux measurements of CO_2_ and CH_4_ above project and control (including both unmanipulated and disturbance) areas can give an estimate of decay rate and emissions. However, emission of gas through fissures may not be captured by surface infrastructure. Given these uncertainties, projects using open systems should continuously measure CH_4_ and CO_2_ within the chamber and conduct periodic sampling from replicated gas collection wells to provide more evidence as to the status of the storage and cap performance (i.e., highlight when breaches may be occurring). Isotopic composition of CH_4_ within the chamber can additionally be compared against surface samples^288^ to quantify the extent of microbial oxidation within the vault cover.

For bio-oil injection into geologic storage sites, there exists significant gaps in research regarding measurement and monitoring of bioproducts. Currently, registries require industrial bio-oil storage projects to MMRV geologic properties and wells prior to, during, and post injection to ensure durable storage conditions are initially met and being maintained. These wells are typically required to have Underground Injection Control (UIC) permits and must characterize the confining system including the well mechanical integrity, reservoir lithology, minerology, porosity, and permeability to ensure durability (e.g.,^289–291^ ).

While measurement and monitoring approaches including geophysical logging methods have been extensively researched and utilized for decades in waste management, CO_2_ storage, and oil and gas sectors (e.g., ASTM Standards D5753, D7400/D7400M, and D5777-18), there is still a need to understand the relevance and application of these technologies for bio-oil storage given the vastly different properties of bio-oil. For example, borehole NMR^292^ and computed tomography approaches^293^ could potentially be used to measure properties of geological storage sites and subsequently monitor bio-oil injections to understand plume behavior and ensure durable reservoir storage; however, it is not known if this is possible given the chemical properties of bio-oil.

On the other hand, standard practices that would still be relevant to measure and monitor for geologic durability include on site gas monitoring. Gas detectors (GC, NDIR, etc.) are required to analyze gases (i.e., CO_2_, CH_4_, etc.) on site during and post injection to ensure there are no leakage or reversal processes contributing to increased gas emissions relative to baseline measurements obtained prior to project deployments. Analysis of reservoir brine (elements, pH, etc.) will also be essential to monitor reversal processes from any geologic storage processes.^294^ At this time, it is essential to characterize the storage sites prior to injection to ensure durability requirements would be met, particularly to meet standards and conform to well regulations, but this data combined with continued monitoring of site properties will be essential for understanding long-term storage processes and predicting durability.

## 7 Conclusions

Nonenergy BiCRS is an emerging field that may have an important role to play in the near-term, either in the interim before higher and better uses of the biomass materialize (i.e., from a circularity or ecosystem services perspective) or as an improvement from the counterfactual treatment of stranded liability biomass, such as that from wildfire mitigation thinning treatments. In this review, we address the current state of knowledge about durability considerations for five nonenergy BiCRS product “forms” under several terrestrial, storage-only “fates.”

Despite the existing research and the availability of analytical methods, the long-term validation of durability claims remains a research frontier across nonenergy BiCRS pathways. The storage of atmospherically derived carbon must be durable over human-relevant time scales to be considered CDR, but the stated durability time scales for BiCRS approaches range widely from contractual standards (10 to >100 years) to project developers (1 year up to 10 million years).^6^ Nonenergy BiCRS projects have not been implemented over time scales long enough to be robustly assessed directly. Field and lab-based studies and existing BiCRS implementations can provide requisite data to calculate decay rates that can be combined with modeling techniques to forecast durability. Biogenic carbon storage analogs on both historical and geological time scales (i.e., wood waste landfills, oil reservoirs, etc.) can further illustrate potential durability. However, long-term storage conditions cannot be absolutely guaranteed in perpetuity due to geophysical events (both recurrent and stochastic) or biotic disturbance.

The time scales of high-risk disturbance regimes that could result in reversal vary widely across BiCRS approaches. For example, near-surface raw biomass burial is more likely to be reversed on a shorter time scale from disturbances such as land use change resulting in excavation or fire than deep geologic bio-oil injection, which could potentially be disturbed by tectonic activity. While prudent feedstock selection, implementation design, and project management can help mitigate the risk of reversal, diligent MMRV for any BiCRS implementation is necessary to continue to build our understanding and modeling capabilities of BiCRS durability. We expect that as technologies and methodologies continue to be developed for assessing chemical recalcitrance, our understanding of carbon product characterization and fate will evolve as well. In addition, specific durability research questions for individual nonenergy BiCRS approaches should be addressed, and we outline pressing research frontiers below.

For the pathway involving dry storage of raw biomass, more research is necessary to integrate findings across pedology, microbiology, and materials science. While soil relative humidity and porewater interactions have been studied in soil matrices and with landfills, further work is necessary to quantify relative humidity, and therefore *a*_*w*_, in the context of storage chambers with significant dry, nonsoil material. Soil relative humidity is a function of soil water potential which includes matric potential, osmotic potential, gravitational potential, and pressure potential.^199^ Matric potential arises from the attraction of water to most surfaces through van der Waals forces, but the strength of this is highly dependent on the characteristics and structure of the solid particles comprising the medium in question. Future research should study the evolution of vault relative humidity and biomass moisture content over time after the introduction of biomass at a quantified initial moisture content. For scenarios that modify osmotic potential to achieve a low *a*_*w*_, further research is necessary to understand how different solute types and cellular adaptation methods either prohibit biotic activity or allow biota to survive under the given conditions and the associated decay pathways. Thermal, moisture and light-mediated reversal mechanisms could also be elucidated for biomass and bioproducts using laboratory technologies traditionally designed for synthetic materials such as the NIST SPHERE.^295^ Finally, if dry biomass storage implementations are going to rely on engineered encasement barriers, further research on the long-term performance of the barrier under field conditions and over decades (at a minimum) is essential.

For any belowground burial approaches, soil disturbance via excavation and vault construction (including compaction) would alter the soil pore structure which could affect water and gas transport. Initial modeling based on predisturbance soil measurements may not capture the changes in cover permeability resulting from the implementation, thus resulting in different storage conditions than expected. Further research on the design, implementation, and deployment of cost-effective biocovers is required to answer questions around CH_4_ oxidation by methanotrophs in the cover and the relationships between chamber conditions (i.e., temperatures, moisture, pH, nutrient availability) and decay rates. For anoxic storage of biochar, BMP tests have never been completed and should be conducted for a variety of biochar feedstocks and processing conditions. These tests will fill a valuable knowledge gap regarding the maximum potential CH_4_ production associated with anaerobic decay of biochar. For anoxic storage of both raw biomass and biochar, it will be critical to track long-term performance of the cover, oxygen concentrations within the burial chamber, and have MMRV systems set up that can indicate early warning signs of failure.

Significant unknowns remain for biomass slurry injection. These include questions about the biogeochemical feedbacks between the biomass, nonbiomass slurry components, biota, and storage environment. Further research is required to understand and optimize slurry formation for successful injection. Safety concerns include potential leakage, induced seismicity, groundwater contamination, and microbial risks triggered by injecting organic material into deep formations. Critical research is needed to understand long-term biogeochemical stability, geo-mechanical impacts, and robust monitoring strategies to ensure permanence and environmental safety.

Finally, there are still unknowns to explore regarding bio-oil phase separation, the proportion of carbon in each phase, and bio-oil storage chemical interactions in geologic reservoirs. For example, mineralogical reactions between bio-oil and rock need to be investigated to understand the potential for dissolution or precipitation which may affect porosity and permeability, as well as compositions of potentially mobile liquids. Trace elements, including transition metals, may be leached from thin grain-boundary or grain-coating phases in sandstones, such as hematite and clays, and partitioned in the aqueous and organic phases of bio-oil. More research on reactions between bio-oil, rock, and resident brine is needed to better understand these reactions, phase dissociation of the bio-oil, partitioning of elements between the phases, the effects of temperature, pressure, oxygen fugacity, and other factors. Experiments that simulate reactive transport of bio-oil through rock and resident brine as these reactions occur and phases dissociate, will be particularly valuable for large-scale predictions of mobility of reaction-induced bio-oil products, and the spatial patterns of mineral dissolution and precipitation in a system of multiple evolving phases. Additionally, further characterization of bio-oil density, including the evolution of density over time under storage-relevant conditions, can inform our understanding as to if and how bio-oil will migrate from an injection site. Lastly, there is still a need to utilize and develop appropriate well- and plume-monitoring technologies for long-term bio-oil storage.

While unknowns still exist, the magnitude of the associated risks and difficulty in assessing and mitigating them ranges widely across nonenergy BiCRS pathways. As outlined in this review, durability metrics for raw and thermochemically converted biomass combined with diligent, scientifically informed storage design and MMRV can steward nonenergy BiCRS pathways as durable CDR in the near-term.

## Appendix: Key Concepts for High-Quality CDR

For any CDR approach to deliver meaningful benefits, the integrity of the project activity must be evaluated—not only the *durability* of the carbon stored. Below we summarize several foundational criteria that must be met across all BiCRS methods discussed in this review.

**Durability.** The expected length of time that atmospherically derived carbon remains stored and isolated from the atmosphere under a given carbon product form and storage fate. It reflects both the intrinsic chemical recalcitrance of the stored carbon to biotic or abiotic decay and the effectiveness of the environmental or engineered controls that suppress re-emission.

**Additionality**. The removal would not have occurred in the absence of the project. This includes demonstrating that carbon removal is not already legally required (regulatory additionality), financially viable without carbon credit revenue (financial additionality), or part of existing business-as-usual operations (carbon additionality).

**Sustainable Biomass Sourcing**. Feedstock must be sourced in ways that protect ecosystems, water, biodiversity, and food security and promote long-term resource availability and environmental and societal health. This includes avoiding overharvesting, soil degradation, and displacement of native vegetation, as well as accounting for emissions from harvesting, collection, and transport.

**Land Use Change**. Projects must avoid direct or indirect land use change that results in carbon emissions or biodiversity loss. For example, converting forests or grasslands into biomass plantations can undermine or even negate net removals. Land use impacts must be evaluated across multiple spatial and temporal scales.

**Counterfactual or Baseline Biomass Use**. High-integrity CDR requires a robust assessment of what would have happened to the biomass in the absence of the project. If biomass would have otherwise been left to decay, burned, or used in other carbon-neutral or carbon-emitting pathways, those emissions must be accurately quantified and incorporated into lifecycle accounting. Counterfactual analysis is especially critical for nonenergy BiCRS pathways because carbon storage is the *sole* climate benefit delivered; thus, removals are only real and additional if the biomass would have released CO_2_ under business-as-usual conditions, rather than being used in ways that already displace fossil emissions or embody long-lived carbon.

**Market Leakage**. Projects should evaluate the risk of indirect emissions caused by displacement effects. For instance, diverting forestry residues from local energy production or mulch markets may create demand for replacement material, resulting in upstream emissions. Leakage must be minimized, monitored, and disclosed.

**Measurement, Monitoring, Reporting, and Verification** (MMRV) refers to the set of practices required to accurately quantify carbon removed, track stored carbon over time, transparently document project performance, and independently confirm that claimed outcomes are real. High-quality CDR requires MMRV systems that are scientifically robust, transparent, and proportionate to the risks of reversal. This includes using validated measurement methods, continuous or risk-appropriate monitoring, standardized reporting protocols, and third-party verification to ensure that durability claims and credited climate benefits are credible and reproducible.

## References

[1] de Coninck, H. ; Revi, A. ; Babiker, M. ; Bertoldi, P. ; Buckeridge, M. ; Cartwright, A. ; Dong, W. ; Ford, J. ; Fuss, S. ; Strengthening and Implementing the Global Response. In Global Warming of 1.5°C. An IPCC Special Report on the impacts of global warming of 1.5°C above pre-industrial levels and related global greenhouse gas emission pathways, in the context of strengthening the global response to the threat of climate change, sustainable development, and efforts to eradicate poverty; Masson-Delmotte, V. , Zhai, P. ; Pörtner, H.-O. , Roberts, D. , Skea, J. , Shukla, P. R. , Pirani, A. , Moufouma-Okia, W. , Péan, C. , Pidcock, R. , Connors, S. , Matthews, J. B. R. , Chen, Y. , Zhou, X. , Gomis, M. I. , Lonnoy, E. , Maycock, T. , Tignor, M. , Waterfield, T. , Eds.;Cambridge University Press: Cambridge, UK and New York, NY, USA, 2018; pp 313–444. 10.1017/9781009157940.006.

[2] IPCC . Annex I: Glossary, in Global Warming of 1.5°C: IPCC Special Report on Impacts of Global Warming of 1.5°C above Pre-industrial Levels in Context of Strengthening Response to Climate Change, Sustainable Development, and Efforts to Eradicate Poverty. Intergovernmental Panel on Climate, Ed.; Cambridge University Press: Cambridge, 2022; pp 541–562. 10.1017/9781009157940.008.

[3] Smith, S. M. ; Geden, O. ; Gidden, M. J. ; Lamb, W. F. ; Nemet, G. F. ; Minx, J. C. ; Buck, H. ; Burke, J. ; Cox, E. ; Edwards, M. R. ; Fuss, S. ; Johnstone, I. ; Müller-Hansen, F. ; Pongratz, J. ; Probst, B. S. ; Roe, S. ; Schenuit, F. ; Schulte, I. ; Vaughan, N. E. The State of Carbon Dioxide Removal, 2nd ed., 2024. https://osf.io/f85qj/.

[4] Smith, S. ; Gregory, O. G. ; Nemet, F. ; Gidden, M. ; Lamb, W. F. ; Powis, C. ; Bellamy, R. ; Callaghan, M. ; Cowie, A. ; Cox, E. ; State of Carbon Dioxide Removal, 1st ed., 2023.

[5] Mistry, K. ; Bahar; Baker, T. ; Ponce de Leon, P. ; Dewar, A. ; Sims, A. The Time for Carbon Removal Has Come; Boston Consulting Group, 2023. Report. Retrieved from: https://web-assets.bcg.com/.

[6] Arcusa S., Hagood E. (2025). Definitions and mechanisms
for managing durability
and reversals in standards and procurers of carbon dioxide removal. Mitigation and Adaptation Strategies for Global Change.

[7] Bunsen F., Nissen C., Hauck J. (2024). The Impact
of Recent Climate Change
on the Global Ocean Carbon Sink. Geophys. Res.
Lett..

[8] MacCarthy J., Tyukavina A., Weisse M. J., Harris N., Glen E. (2024). Extreme wildfires
in Canada and their contribution to global loss in tree cover and
carbon emissions in 2023. Global Change Biology.

[9] Soong J. L., Castanha C., Hicks Pries C. E., Ofiti N., Porras R. C., Riley W. J., Schmidt M.W. I., Torn M. S. (2021). Five years of whole-soil
warming led to loss of subsoil carbon stocks and increased CO2 efflux. Science Advances.

[10] USFS . Future of America’s Forest and Rangelands; U.S. Department of Agriculture, Forest Service, 2023. 10.2737/WO-GTR-102.

[11] Lal R. (2003). Soil erosion
and the global carbon budget. Environ. Int..

[12] Loehman R. A., Reinhardt E., Riley K. L. (2014). Wildland fire emissions, carbon,
and climate: Seeing the forest and the trees - A cross-scale assessment
of wildfire and carbon dynamics in fire-prone, forested ecosystems. Forest Ecology and Management.

[13] Sandalow, D. ; Aines, R. ; Friedmann, J. ; McCormick, C. ; Sanchez, D. L. Biomass Carbon Removal and Storage (BiRCS) Roadmap. 2021: United States. https://www.osti.gov/biblio/1763937.

[14] Woodall, C. M. ; McCormick, C. F. Assessing the optimal uses of biomass: Carbon and energy price conditions for the Aines Principle to apply. Frontiers in Climate 2022, 4. 10.3389/fclim.2022.993230.

[15] Pett-Ridge, J. ; Kuebbing, S. ; Mayer, A. C. ; Hovorka, S. ; Pilorgé, H. ; Baker, S. E. ; Pang, S. H. ; Scown, C. D. ; Mayfield, K. K. ; Wong, A. A. ; Roads to Removal: Options for Carbon Dioxide Removal in the United States. 2023. United States. https://www.osti.gov/biblio/2301853.

[16] Yablonovitch E., Deckman H. W. (2023). Scalable, economical,
and stable sequestration of agricultural
fixed carbon. Proc. Natl. Acad. Sci. U.S.A..

[17] Janda, A. Exaquest Carbon Wood drying and preservation research results for January, 2022 - present. Research Report, 2022.

[18] Jovine, R. Method of Carbon Sequestration US 8,278,082 B2. U. S. P. a. T. Office, 2012.

[19] Zeng N., King A. W., Zaitchik B., Wullschleger S. D., Gregg J., Wang S., Kirk-Davidoff D. (2013). Carbon sequestration
via wood harvest and storage: An assessment of its harvest potential. Climatic Change.

[20] Zeng N., Hausmann H. (2022). Wood Vault: remove
atmospheric CO2 with trees, store
wood for carbon sequestration for now and as biomass, bioenergy and
carbon reserve for the future. Carbon Balance
and Management.

[21] Soeherman J. K., Jones A. J., Dauenhauer P. J. (2023). Overcoming
the Entropy Penalty of
Direct Air Capture for Efficient Gigatonne Removal of Carbon Dioxide. ACS Engineering Au.

[22] Charm Industrial . Bio-Oil Sequestration as a Viable CDR Pathway (Whitepaper), 2025. https://charmindustrial.com/bio-oil-injection-whitepaper.

[23] Isometric . Subsurface Biomass Carbon Removal and Storage: BiCRS Protocol, version 1.0, 2024.

[24] Murdoch L. C., Germanovich L. N., Slack W. W., Carbajales-Dale M., Knight D., Moak R., Laffaille C., DeWolf S., Roudini S. (2023). Shallow Geologic Storage
of Carbon
to Remove Atmospheric CO2 and Reduce Flood Risk. Environ. Sci. Technol..

[25] Adair E. C., Parton W. J., Del Grosso S. J., Silver W. L., Harmon M. E., Hall S. A., Burke I. C., Hart S. C. (2008). Simple three-pool
model accurately describes patterns of long-term litter decomposition
in diverse climates. Global Change Biology.

[26] Austin A. T., Ballaré C. L. (2010). Dual role of lignin in plant litter decomposition in
terrestrial ecosystems. Proc. Natl. Acad. Sci.
U. S. A..

[27] Martínez A. T., Rencoret J., Nieto L., Jiménez-Barbero J., Gutiérrez A., del Río J. C. (2011). Selective lignin and polysaccharide
removal in natural fungal decay of wood as evidenced by in situ structural
analyses. Environmental Microbiology.

[28] Crombie K., Mašek O., Sohi S. P., Brownsort P., Cross A. (2013). The effect of pyrolysis conditions on biochar stability as determined
by three methods. GCB Bioenergy.

[29] Tomczyk A., Sokołowska Z., Boguta P. (2020). Biochar physicochemical properties:
pyrolysis temperature and feedstock kind effects. Reviews in Environmental Science and Bio/Technology.

[30] Brischke C., Rapp A. O. (2008). Dose-response relationships
between wood moisture content,
wood temperature and fungal decay determined for 23 European field
test sites. Wood Science and Technology.

[31] Cogulet A., Blanchet P., Landry V. (2016). Wood degradation under UV irradiation:
A lignin characterization. Journal of Photochemistry
and Photobiology B: Biology.

[32] Sanei H., Rudra A., Przyswitt Z. M. M., Kousted S., Sindlev M. B., Zheng X., Nielsen S. B., Petersen H. I. (2024). Assessing biochar’s
permanence: An inertinite benchmark. International
Journal of Coal Geology.

[33] Bai Z., Ma Q., Dai Y., Yuan H., Ye J., Yu W. (2017). Spatial Heterogeneity
of SOM Concentrations Associated with White-rot Versus Brown-rot Wood
Decay. Sci. Rep..

[34] Ulyshen M. D. (2016). Wood decomposition
as influenced by invertebrates. Biological Reviews.

[35] Li H., Dong X., da Silva E. B., de Oliveira L. M., Chen Y., Ma L. Q. (2017). Mechanisms of metal
sorption by biochars:
Biochar characteristics and modifications. Chemosphere.

[36] Kelemen, P. ; Benson, S. M. ; Pilorgé, H. ; Psarras, P. ; Wilcox, J. , An Overview of the Status and Challenges of CO2 Storage in Minerals and Geological Formations. Frontiers in Climate 2019, 1. 10.3389/fclim.2019.00009.

[37] Budai, A. ; Zimmerman, A. ; Cowie, A. ; Webber, J. ; Singh, B. P. ; Glaser, B. ; Masiello, C. ; Andersson, D. ; Lehmann, J. ; Camps Arbestain, M. ; Biochar Carbon Stability Test Method: An assessment of methods to determine biochar carbon stability. International Biochar Initiative 2013. 10.13140/RG.2.2.16359.42402.

[38] Enders A., Hanley K., Whitman T., Joseph S., Lehmann J. (2012). Characterization
of biochars to evaluate recalcitrance and agronomic performance. Bioresour. Technol..

[39] Biochar for Environmental Management: Science, Technology and Implementation, 2nd ed.; Lehmann, J. ; Joseph, S. , Eds.; Routledge, 2015. 10.4324/9780203762264.

[40] Lourenço, A. ; Pereira, H. Compositional Variability of Lignin in Biomass. In Lignin - Trends and Applications; Poletto, M. , Ed.; IntechOpen, 2017. 10.5772/intechopen.71208.

[41] Vassilev S. V., Baxter D., Andersen L. K., Vassileva C. G. (2010). An overview
of the chemical composition of biomass. Fuel.

[42] Li D., Yang J., Pak S., Zeng M., Sun J., Yu S., He Y., Li C. (2022). PuC3H35 confers drought tolerance
by enhancing lignin and proanthocyanidin biosynthesis in the roots
of Populus ussuriensis. New Phytologist.

[43] Bryant, N. ; Muchero, W. ; Weber, R. A. ; Barros, J. ; Chen, J.-G. ; Tschaplinski, T. J. ; Pu, Y. ; Ragauskas, A. J. Cell wall response of field grown Populus to Septoria infection. Frontiers in Plant Science 2023, 14. 10.3389/fpls.2023.1089011. PMC10282658 37351208

[44] Mazarei M., Harman-Ware A. E., Pendergast T. H., Shrestha V., Xu Y., Piasecki C., Millwood R. J., Devos K. M., Stewart C. N. (2025). Variation
in Biomass Yield and Cell Wall Composition in Switchgrass Natural
Variants Under Two Nitrogen Regimes. BioEnergy
Research.

[45] Adhikari, S. ; Nam, H. ; Chakraborty, J. P. Chapter 8 - Conversion of Solid Wastes to Fuels and Chemicals Through Pyrolysis. In Waste Biorefinery; Bhaskar, T. , Pandey, A. , Mohan, S. V. , Lee, D.-J. , Khanal, S. K. , Eds.; Elsevier, 2018; pp 239–263. 10.1016/B978-0-444-63992-9.00008-2.

[46] Bentsen N. S., Felby C., Thorsen B. J. (2014). Agricultural
residue production and
potentials for energy and materials services. Prog. Energy Combust. Sci..

[47] Praspaliauskas M., Pedišius N., Čepauskiene D., Valantinavičius M. (2020). Study of chemical
composition of agricultural residues from various agro-mass types. Biomass Conversion and Biorefinery.

[48] Raj T., Kapoor M., Gaur R., Christopher J., Lamba B., Tuli D. K., Kumar R. (2015). Physical and
Chemical
Characterization of Various Indian Agriculture Residues for Biofuels
Production. Energy Fuels.

[49] Santana-Méridas O., González-Coloma A., Sánchez-Vioque R. (2012). Agricultural
residues as a source of bioactive natural products. Phytochemistry Reviews.

[50] Caicedo M., Barros J., Ordás B. (2016). Redefining
Agricultural Residues
as Bioenergy Feedstocks. Materials.

[51] Gholz H. L., Wedin D. A., Smitherman S. M., Harmon M. E., Parton W. J. (2000). Long-term
dynamics of pine and hardwood litter in contrasting environments:
toward a global model of decomposition. Global
Change Biology.

[52] Harman-Ware A. E., Sparks S., Addison B., Kalluri U. C. (2021). Importance of suberin
biopolymer in plant function, contributions to soil organic carbon
and in the production of bio-derived energy and materials. Biotechnol Biofuels.

[53] Harman-Ware A. E. (2020). Conversion
of Terpenes to Chemicals and Related Products. Chemical Catalysts for Biomass Upgrading.

[54] Weaver J. M., Lohrey G., Tomasi P., Dyer J. M., Jenks M. A., Feldmann K. A. (2018). Cuticular wax variants in a population
of switchgrass
(Panicum virgatum L.). Industrial Crops and
Products.

[55] Puri, L. ; Hu, Y. ; Naterer, G. Critical review of the role of ash content and composition in biomass pyrolysis [Review]. Frontiers in Fuels 2024, 2. 10.3389/ffuel.2024.1378361.

[56] Barker J., Voorhis J., Crotty S. M. (2025). Assessing
costs and constraints of
forest residue disposal by pile burning. Frontiers
in Forests and Global Change.

[57] Rowell, R. M. Handbook of Wood Chemistry and Wood Composites, 2nd ed.; CRC Press, 2012. 10.1201/b12487.

[58] Balk M., Sofia P., Neffe A. T., Tirelli N. (2023). Lignin, the Lignification
Process, and Advanced, Lignin-Based Materials. Int. J. Mol. Sci..

[59] Sjöström, E. Wood Chemistry, 2nd ed.; Academic Press: San Diego, CA, USA, 1993; pp 90–108.

[60] Lehr M., Miltner M., Friedl A. (2021). Removal of wood extractives as pulp
(pre-)­treatment: a technological review. SN
Applied Sciences.

[61] Huang F., Singh P. M., Ragauskas A. J. (2011). Characterization
of Milled Wood Lignin
(MWL) in Loblolly Pine Stem Wood, Residue, and Bark. J. Agric. Food Chem..

[62] Wawro A., Jakubowski J., Gieparda W., Pilarek Z., Łacka A. (2023). Potential
of Pine Needle Biomass for Bioethanol Production. Energies.

[63] Berg B., Wessen B., Ekbohm G., Berg B., Wessen B. (1982). Nitrogen Level
and Decomposition in Scots Pine Needle Litter. Oikos.

[64] Jan A., Shah J., Khan F. U., Rahman A. U. (2006). Short Communication
Investigation of Pine Needles for Pulp/Paper Industry. Biological Sciences - PJSIR.

[65] Sanchez F. G. (2001). Loblolly
pine needle decomposition and nutrient dynamics as affected by irrigation,
fertilization, and substrate quality. Forest
Ecology and Management.

[66] Theander, O. Cellulose, Hemicellulose and Extractives. In Fundamentals of Thermochemical Biomass Conversion; Overend, R. P. , Milne, T. A. , Mudge, L. K. , Eds.; Springer, Dordrecht, 1985. 10.1007/978-94-009-4932-4_2.

[67] Erdenebileg E., Wang C., Ye X., Cui Q., Du J., Huang Z., Liu G., Cornelissen J. H. C. (2020). Multiple
abiotic and biotic drivers of long-term wood decomposition within
and among species in the semi-arid inland dunes: A dual role for stem
diameter. Functional Ecology.

[68] Fukasawa Y., Kaga K. (2020). Effects of wood resource
size and decomposition on hyphal outgrowth
of a cord-forming basidiomycete, Phanerochaete velutina. Sci. Rep.

[69] Fukasawa Y., Kaga K. (2022). Surface Area of Wood Influences the
Effects of Fungal Interspecific
Interaction on Wood Decomposition-A Case Study Based on Pinus densiflora
and Selected White Rot Fungi. J. Fungi (Basel).

[70] Sorz J., Hietz P. (2006). Gas diffusion through
wood: Implications for oxygen supply. Trees.

[71] Hu Z., Michaletz S. T., Johnson D. J., McDowell N. G., Huang Z., Zhou X., Xu C. (2018). Traits drive global wood decomposition
rates more than climate. Global Change Biology.

[72] Firmansyah F., Park I., Corona M., Aphale O., Ahuja A., Johnston M., Thyberg K. L., Hewitt E., Tonjes D. J. (2024). Variation
in municipal solid waste generation and management across time and
space. Resources, Conservation and Recycling.

[73] Burnley S. J. (2007). A review
of municipal solid waste composition in the United Kingdom. Waste Management.

[74] Funk, K. ; Milford, J. ; Simpkins, T. Chapter 19 - Waste not, want not: analyzing the economic and environmental viability of waste-to-energy technology for site-specific optimization of renewable energy options. In Bioenergy (Second ed.); Dahiya, A. , Ed.; Academic Press, 2020; pp 385–423. 10.1016/B978-0-12-815497-7.00019-1.

[75] Olawade D. B., Fapohunda O., Wada O. Z., Usman S. O., Ige A. O., Ajisafe O., Oladapo B. I. (2024). Smart waste management: A paradigm
shift enabled by artificial intelligence. Waste
Management Bulletin.

[76] Razzak S. A. (2024). Municipal
solid and plastic waste derived high-performance biochar production:
A comprehensive review. J. Anal. Appl. Pyrolysis.

[77] Chapman E. A., Thomsen H. C., Tulloch S., Correia P. M. P., Luo G., Najafi J., DeHaan L. R., Crews T. E., Olsson L., Lundquist P. O. (2022). Perennials as Future Grain Crops: Opportunities
and Challenges. Frontiers in plant science.

[78] Wang X., Lu X., Li F., Yang G. (2014). Effects of Temperature and Carbon-Nitrogen
(C/N) Ratio on the Performance of Anaerobic Co-Digestion of Dairy
Manure, Chicken Manure and Rice Straw: Focusing on Ammonia Inhibition. PLoS One.

[79] James J., Page-Dumroese D., Busse M., Palik B., Zhang J., Eaton B., Slesak R., Tirocke J., Kwon H. (2021). Effects of
forest harvesting and biomass removal on soil carbon and nitrogen:
Two complementary meta-analyses. Forest Ecology
and Management.

[80] Hu X., Liu B., Deng Y., Bao X., Yang A., Zhou J. (2019). A novel two-stage
culture strategy used to cultivate Chlorella vulgaris for increasing
the lipid productivity. Sep. Purif. Technol..

[81] Qian P., Schoenau J. J. (2002). Availability of
nitrogen in solid manure amendments
with different C:N ratios. Canadian Journal
of Soil Science.

[82] Ikram, M. ; Mehran, M. ; Minhas, A. ; ur Rehman, H. ; Bakash, M. Z. M. ; Khan, M. W. ; Khan, M. M. S. , El Sabagh, A. ; Rasheed, H. C:N Ratio and Its Importance in Developing Effective Bioenergy Crops. In Forage Crops in the Bioenergy Revolution: From Fields to Fuel; Singhal, R. K. , Indu, El Sabagh, A. , Dwivedi, K. K. , Eds.; Springer Nature Singapore, 2025; pp 259–276. 10.1007/978-981-96-2536-9_14.

[83] Rai M. P., Nigam S., Sharma R. (2013). Response of
growth and fatty acid
compositions of Chlorella pyrenoidosa under mixotrophic cultivation
with acetate and glycerol for bioenergy application. Biomass and Bioenergy.

[84] Addison B., Bu L., Bharadwaj V., Crowley M. F., Harman-Ware A. E., Crowley M. F., Bomble Y. J., Ciesielski P. N. (2024). Atomistic,
macromolecular model of the Populus secondary cell wall informed by
solid-state NMR. Science Advances.

[85] Barlaz, M. Microbiology of Solid Waste Landfills. In Palmisano, A. B. ; Morton (Ed.), Microbiology of Solid Waste (pp 42). CRC Press, 1996.

[86] Zoghlami, A. ; Paës, G. , Lignocellulosic Biomass: Understanding Recalcitrance and Predicting Hydrolysis. Frontiers in Chemistry 2019, 7. 10.3389/fchem.2019.00874. PMC6930145 31921787

[87] Katahira, R. ; Elder, T. J. ; Beckham, G. T. A Brief Introduction to Lignin Structure. In Lignin Valorization: Emerging Approaches; Beckham, G. T. , Ed.; The Royal Society of Chemistry, 2018. 10.1039/9781788010351-00001.

[88] Vanholme R., Demedts B., Morreel K., Ralph J., Boerjan W. (2010). Lignin Biosynthesis
and Structure. Plant Physiology.

[89] Yoo C. G., Yang Y., Pu Y., Meng X., Muchero W., Yee K. L., Thompson O. A., Rodriguez M., Bali G., Engle (2017). Insights of biomass
recalcitrance in natural Populus
trichocarpa variants for biomass conversion. Green Chem..

[90] Chen F., Dixon R. A. (2007). Lignin modification improves fermentable sugar yields
for biofuel production. Nat. Biotechnol..

[91] Aerts R. (1997). Climate, Leaf
Litter Chemistry and Leaf Litter Decomposition in Terrestrial Ecosystems:
A Triangular Relationship. Oikos.

[92] Perez-Harguindeguy N., Diaz S., Vendramini F., Gurvich D. E., Cingolani A. M., Giorgis M. A., Cabido M. (2007). Direct and
indirect effects of climate
on decomposition in native ecosystems from central Argentina. Austral Ecology.

[93] Isahak W. N. R. W., Hisham M. W. M., Yarmo M. A., Yun Hin T.-y. (2012). A review on bio-oil
production from biomass by using pyrolysis method. Renewable and Sustainable Energy Reviews.

[94] Tumuluru, J. S. ; Ghiasi, B. ; Soelberg, N. R. ; Sokhansanj, S. Biomass Torrefaction Process, Product Properties, Reactor Types, and Moving Bed Reactor Design Concepts. Frontiers in Energy Research 2021, 9. 10.3389/fenrg.2021.728140.

[95] Schmidt H. P. (2025). Biochar Permanence—A Policy Commentary. Global Change Biology Bioenergy.

[96] Wood R., Mašek O., Erastova V. (2024). Developing a molecular-level understanding
of biochar materials using public characterization data. Cell Reports Physical Science.

[97] Rodrigues L., Budai A., Elsgaard L., Hardy B., Keel S. G., Mondini C., Plaza C., Leifeld J. (2023). The importance of biochar
quality and pyrolysis yield for soil carbon sequestration in practice. European Journal of Soil Science.

[98] Mohan D., Pittman C. U., Steele P. H. (2006). Pyrolysis of Wood/Biomass
for Bio-oil: A Critical Review. Energy Fuels.

[99] Cai J., Rahman M. M., Zhang S., Sarker M., Zhang X., Zhang Y., Yu X., Fini E. H. (2021). Review on Aging
of Bio-Oil from Biomass Pyrolysis and Strategy to Slowing Aging. Energy Fuels.

[100] Werner C., Schmidt H. P., Gerten D., Lucht W., Kammann C. (2018). Biogeochemical potential of biomass
pyrolysis systems
for limiting global warming to 1.5 °C. Environmental Research Letters.

[101] Dubey P., Gnangbe S., Mba Wright M. (2025). Enhancing
carbon removal via scalable on-site pyrolysis and well-plugging systems. Energy Conversion and Management.

[102] Garcia-Nunez J. A., Pelaez-Samaniego M. R., Garcia-Perez M. E., Fonts I., Abrego J., Westerhof R. J. M., Garcia-Perez M. (2017). Historical Developments of Pyrolysis Reactors: A Review. Energy Fuels.

[103] Woolf D., Lehmann J., Joseph S., Campbell C., Christo F. C., Angenent L. T. (2017). An open-source biomass
pyrolysis
reactor. Biofuels, Bioproducts and Biorefining.

[104] Zheng Y., Tao L., Yang X., Huang Y., Liu C., Gu J., Zheng Z. (2017). Effect of
the torrefaction temperature
on the structural properties and pyrolysis behavior of biomass. BioResources.

[105] Chen W.-H., Peng J., Bi X. T. (2015). A state-of-the-art
review of biomass torrefaction, densification and applications. Renew. Sustain. Energy Rev..

[106] Lin S.-L., Zhang H., Chen W.-H., Song M., Kwon E. E. (2023). Low-temperature biochar production
from torrefaction
for wastewater treatment: A review. Bioresour.
Technol..

[107] Li S., Harris S., Anandhi A., Chen G. (2019). Predicting biochar
properties and functions based on feedstock and pyrolysis temperature:
A review and data syntheses. Journal of Cleaner
Production.

[108] DeGroot W. F., Shafizadeh F. (1984). Kinetics of gasification of Douglas
Fir and Cottonwood chars by carbon dioxide. Fuel.

[109] Boerrigter, H. ; Rauch, R. Review of applications of gases from biomass gasification. Technical Report. ECN-RX-06-066, 2006. Energy research Centre of the Netherlands ECN, Petten (Netherlands).

[110] Basu, P. Chapter 7 - Gasification Theory. In Biomass Gasification, Pyrolysis and Torrefaction (Third ed.); Basu, P. , Ed.; Academic Press, 2018; pp 211–262. 10.1016/B978-0-12-812992-0.00007-8.

[111] Ahmad A. A., Zawawi N. A., Kasim F. H., Inayat A., Khasri A. (2016). Assessing the gasification performance of biomass:
A review on biomass gasification process conditions, optimization
and economic evaluation. Renewable and Sustainable
Energy Reviews.

[112] Brown R. C. (2021). The Role of Pyrolysis and Gasification in a Carbon
Negative Economy. Processes.

[113] Stolaroff, J. K. ; Pang, S. H. ; Li, W. ; Kirkendall, W. G. ; Goldstein, H. M. ; Aines, R. D. ; Baker, S. E. Transport Cost for Carbon Removal Projects With Biomass and CO2 Storage. Frontiers in Energy Research 2021, 9. 10.3389/fenrg.2021.639943.

[114] Wang S., Wu L., Hu X., Zhang L., Li T., Li C.-Z. (2018). Effects of the Particle
Size and Gasification Atmosphere
on the Changes in the Char Structure during the Gasification of Mallee
Biomass. Energy Fuels.

[115] Woolf D., Lehmann J., Ogle S., Kishimoto-Mo A. W., McConkey B., Baldock J. (2021). Greenhouse Gas Inventory
Model for
Biochar Additions to Soil. Environ. Sci. Technol..

[116] Tan H., Lee C. T., Ong P. Y., Wong K. Y., Bong C. P. C., Li C., Gao Y. (2021). A Review On The Comparison Between
Slow Pyrolysis And Fast Pyrolysis On The Quality Of Lignocellulosic
And Lignin-Based Biochar. IOP Conference Series:
Materials Science and Engineering.

[117] Arriola E., Chen W.-H., Chih Y.-K., De Luna M. D., Show P. L. (2020). Impact of post-torrefaction process
on biochar formation
from wood pellets and self-heating phenomena for production safety. Energy.

[118] Khater E.-S., Bahnasawy A., Hamouda R., Sabahy A., Abbas W., Morsy O. M. (2024). Biochar production under different
pyrolysis temperatures with different types of agricultural wastes. Sci. Rep..

[119] Bridgwater A. V., Meier D., Radlein D. (1999). An overview
of fast
pyrolysis of biomass. Org. Geochem..

[120] Wang G., Dai Y., Yang H., Xiong Q., Wang K., Zhou J., Li Y., Wang S. (2020). A Review of
Recent Advances in Biomass Pyrolysis. Energy
Fuels.

[121] Czernik S., Bridgwater A. V. (2004). Overview
of Applications of Biomass
Fast Pyrolysis Oil. Energy Fuels.

[122] Staš M., Auersvald M., Kejla L., Vrtiška D., Kroufek J., Kubička D. (2020). Quantitative
analysis of pyrolysis
bio-oils: A review. Trends Anal. Chem..

[123] Fryda L., Visser R. (2015). Biochar for Soil Improvement:
Evaluation
of Biochar from Gasification and Slow Pyrolysis. Agriculture.

[124] You S., Ok Y. S., Chen S. S., Tsang D. C. W., Kwon E. E., Lee J., Wang C.-H. (2017). A critical review
on sustainable biochar system through
gasification: Energy and environmental applications. Bioresource.

[125] Demirbas A., Arin G. (2002). An Overview of Biomass
Pyrolysis. Energy Sources.

[126] Zhu C., Krumm C., Facas G. G., Neurock M., Dauenhauer P. J. (2017). Energetics
of cellulose and cyclodextrin glycosidic bond cleavage. Reaction Chemistry & Engineering.

[127] Di Blasi C., Signorelli G., Di Russo C., Rea G. (1999). Product Distribution
from Pyrolysis of Wood and Agricultural Residues. Ind. Eng. Chem. Res..

[128] Fahmi R., Bridgwater A. V., Donnison I., Yates N., Jones J. M. (2008). The effect of lignin
and inorganic species in biomass
on pyrolysis oil yields, quality and stability. Fuel.

[129] Patwardhan P. R., Satrio J. A., Brown R. C., Shanks B. H. (2010). Influence
of inorganic salts on the primary pyrolysis products of cellulose. Bioresour. Technol..

[130] He Z., Zhao A., Liu S., Chen Y., Liu J., Zhao W., Yin M., Dong Q., Zhang J., Zhang G., Bi D. (2024). Preparation
of nitrogen-containing
chemicals from lignocellulosic biomass and nitrogen-rich organic solid
waste by pyrolysis: Characteristics, reaction mechanisms, and feedstock
interactions. Chemical Engineering Journal.

[131] Hassan E. M., Yu F., Ingram L., Steele P. (2009). The Potential
Use of Whole-tree Biomass for Bio-oil Fuels. Energy Sources, Part A: Recovery, Utilization, and Environmental
Effects.

[132] Carpenter D., Westover T. L., Czernik S., Jablonski W. (2014). Biomass feedstocks
for renewable fuel production: a review of the impacts of feedstock
and pretreatment on the yield and product distribution of fast pyrolysis
bio-oils and vapors. Green Chem..

[133] Debiagi P., Gentile G., Cuoci A., Frassoldati A., Ranzi E., Faravelli T. (2018). A predictive
model of biochar formation
and characterization. Journal of Analytical
and Applied Pyrolysis.

[134] Ong H. C., Yu K. L., Chen W.-H., Pillejera M. K., Bi X., Tran K.-Q., Pétrissans A., Pétrissans M. (2021). Variation
of lignocellulosic biomass structure from torrefaction: A critical
review. Renewable and Sustainable Energy Reviews.

[135] Leng L., Huang H., Li H., Li J., Zhou W. (2019). Biochar stability assessment methods: A review. Science of The Total Environment.

[136] Fawzy S., Osman A. I., Yang H., Doran J., Rooney D. W. (2021). Industrial biochar systems for atmospheric
carbon removal:
a review. Environmental Chemistry Letters.

[137] Spokas K. A. (2010). Review
of the stability of biochar in soils: predictability
of O:C molar ratios. Carbon Management.

[138] Wijitkosum S., Sriburi T. (2023). Aromaticity,
polarity, and longevity
of biochar derived from disposable bamboo chopsticks waste for environmental
application. Heliyon.

[139] ISO . Methods for the petrographic analysis of coals. In Part 5: Method of determining microscopically the reflectance of vitrinite, Vol. 7404-5:2009.

[140] Murray J., Keith A., Singh B. (2015). The stability of low-
and high-ash biochars in acidic soils of contrasting mineralogy. Soil Biology and Biochemistry.

[141] Ravi S., Li J., Meng Z., Zhang J., Mohanty S. (2020). Generation, Resuspension, and Transport
of Particulate
Matter From Biochar-Amended Soils: A Potential Health Risk. GeoHealth.

[142] Ravi S., Sharratt B. S., Li J., Olshevski S., Meng Z., Zhang J. (2016). Particulate matter
emissions from
biochar-amended soils as a potential tradeoff to the negative emission
potential. Sci. Rep..

[143] Xie Y., Li C., Chen H., Gao Y., Vancov T., Keen B., Van Zwieten L., Fang Y., Sun X., He Y., Li X., Bolan N., Yang X., Wang H. (2024). Methods for
quantification of biochar in soils: A critical review. CATENA.

[144] Chen M., Wang D., Yang F., Xu X., Xu N., Cao X. (2017). Transport and retention of biochar nanoparticles in
a paddy soil under environmentally-relevant solution chemistry conditions. Environ. Pollut..

[145] Weber K., Quicker P. (2018). Properties of biochar. Fuel.

[146] Chen D., Zhou J., Zhang Q., Zhu X. (2014). Evaluation
methods and research progresses in bio-oil storage stability. Renewable and Sustainable Energy Reviews.

[147] Wang C., Ding H., Zhang Y., Zhu X. (2020). Analysis of
property variation and stability on the aging of bio-oil from fractional
condensation. Renewable Energy.

[148] Blin J., Volle G., Girard P., Bridgwater T., Meier D. (2007). Biodegradability of biomass pyrolysis
oils: Comparison to conventional
petroleum fuels and alternatives fuels in current use. Fuel.

[149] Diebold, J. A Review of the Chemical and Physical Mechanisms of the Storage Stability of Fast Pyrolysis Bio-Oils, 2000. https://docs.nrel.gov/docs/fy00osti/27613.pdf.

[150] Lindfors C., Kuoppala E., Oasmaa A., Solantausta Y., Arpiainen V. (2014). Fractionation of Bio-Oil. Energy
Fuels.

[151] Song Q.-H., Nie J.-Q., Ren M.-G., Guo Q.-X. (2009). Effective
Phase Separation of Biomass Pyrolysis Oils by Adding Aqueous Salt
Solutions. Energy Fuels.

[152] Vitasari C. R., Meindersma G. W., de Haan A. B. (2011). Water extraction
of pyrolysis oil: The first step for the recovery of renewable chemicals. Bioresour. Technol..

[153] Bradford M. A., Berg B., Maynard D. S., Wieder W. R., Wood S. A. (2016). Understanding the dominant controls
on litter decomposition. Journal of Ecology.

[154] Seigle-Murandi F., Guiraud P., Croize J., Falsen E., Eriksson K. L. (1996). Bacteria
Are Omnipresent on Phanerochaete chrysosporium
Burdsall. Appl. Environ. Microbiol..

[155] Pastorelli R., Agnelli A. E., De Meo I., Graziani A., Paletto A., Lagomarsino A. (2017). Analysis of Microbial Diversity and
Greenhouse Gas Production of Decaying Pine Logs. Forests.

[156] Tláskal, V. ; Brabcová, V. ; Větrovský, T. ; Jomura, M. ; López-Mondéjar, R. ; Oliveira Monteiro Lummy, M. ; Saraiva João, P. ; Human Zander, R. ; Cajthaml, T. ; Nunes da Rocha, U. ; Baldrian, P. Complementary Roles of Wood-Inhabiting Fungi and Bacteria Facilitate Deadwood Decomposition. mSystems 2021, 6 (1). 10.1128/mSystems.01078-20. PMC7901482 33436515

[157] Goodell B., Winandy J. E., Morrell J. J. (2020). Fungal
Degradation
of Wood: Emerging Data, New Insights and Changing Perceptions. Coatings.

[158] Zhu, Y. ; Plaza, N. ; Kojima, Y. ; Yoshida, M. ; Zhang, J. ; Jellison, J. ; Pingali, S. V. ; O’Neill, H. ; Goodell, B. Nanostructural Analysis of Enzymatic and Non-enzymatic Brown Rot Fungal Deconstruction of the Lignocellulose Cell Wall. Front. Microbiol. 2020, 11. 10.3389/fmicb.2020.01389. PMC7326796 32670241

[159] Shraddha, Shekher R., Sehgal S., Kamthania M., Kumar A. (2011). Laccase: microbial
sources, production, purification, and potential biotechnological
applications. Enzyme Res..

[160] Lustenhouwer N., Maynard D. S., Bradford M. A., Lindner D. L., Oberle B., Zanne A. E., Crowther T. W. (2020). A trait-based understanding
of wood decomposition by fungi. Proc. Natl.
Acad. Sci. U. S. A..

[161] Hamed S. A. M. (2013). In-vitro studies on wood degradation
in soil by soft-rot
fungi: Aspergillus niger and Penicillium chrysogenum. International Biodeterioration & Biodegradation.

[162] Odier E., Janin G., Monties B. (1981). Poplar lignin
decomposition
by gram-negative aerobic bacteria. Appl. Environ.
Microbiol..

[163] Xu R., Zhang K., Liu P., Han H., Zhao S., Kakade A., Khan A., Du D., Li X. (2018). Lignin depolymerization
and utilization by bacteria. Bioresour. Technol..

[164] Hilger, H. ; Barlaz, M. A. Anaerobic Decomposition of Refuse in Landfills and Methane Oxidation in Landfill Covers. In Manual of Environmental Microbiology; Hurst, C.J. , Crawford, R.L. , Garland, J.L. , Lipson, D.A. , Mills, A.L. , Stetzenbach, L.D. , Eds.; 2007. 10.1128/9781555815882.ch67.

[165] Joly F.-X., Scherer-Lorenzen M., Hättenschwiler S. (2023). Resolving
the intricate role of climate in litter decomposition. Nature Ecology & Evolution.

[166] George B., Suttie E., Merlin A., Deglise X. (2005). Photodegradation
and photostabilisation of wood - the state of the art. Polym. Degrad. Stab..

[167] Suttie, E. The photodegradation of wood - Defining activation spectra for colour change. Fifth International Wood Coating Congress: “Enhancing service life″; Prague/Czech Republic, 2006. 7.

[168] Day T. A., Guénon R., Ruhland C. T. (2015). Photodegradation
of plant litter in the Sonoran Desert varies by litter type and age. Soil Biology and Biochemistry.

[169] Cordero R. R., Seckmeyer G., Damiani A., Riechelmann S., Rayas J., Labbe F., Laroze D. (2013). The world’s
highest levels of surface UV. Photochemical
& Photobiological Sciences.

[170] Lehmann J., Abiven S., Azzi E. (2024). “
Persistence of Biochar: Mechanisms, Measurements, Predictions.”. Biochar for Environmental Management: Science, Technology
and Implementation.

[171] Li (2020). Evaluation
of biochar properties exposing to solar radiation: A promotion on
surface activities. Chem. Eng. J..

[172] Scott W. (1953). Water Relations of Staphylococcus
Aureus At 30°C. Australian Journal of Biological
Sciences.

[173] Chirife J., Fontana A. J. (2020). Chapter 1: Introduction,
Historical Highlights of Water Activity Research. Water Activity in Foods.

[174] Scott W. J. (1957). Water relations
of food spoilage microorganisms. Adv. Food Res..

[175] Pitt, J.I. ; A.D.H. Fungi and Food Spoilage; Springer Science Business Media, LLC, 2009. 10.1007/978-0-387-92207-2_2.

[176] Pitt, J. I. Xerophilic fungi and the spoilage of foods of plant origin. In Water Relations of Foods; Duckworth, R. B. , Ed.; Academic Press, 1975; pp 273–307.

[177] Stevenson A., Cray J. A., Williams J. P., Santos R., Sahay R., Neuenkirchen N., McClure C. D., Grant I. R., Houghton J. D. R., Quinn J. P. (2015). Is there a common water-activity
limit for the three domains of life?. ISME Journal.

[178] Bittelli, M. ; Campbell, G. S. ; Tomei, F. Soil physics with Python: transport in the soil-plant-atmosphere system; OUP Oxford, 2015.

[179] Chamas A., Moon H., Zheng J., Qiu Y., Tabassum T., Jang J. H., Abu-Omar M., Scott S. L., Suh S. (2020). Degradation
Rates of Plastics in the Environment. ACS Sustainable
Chem. Eng..

[180] Lenz M., Creffield J. W., Evans T. A., Kard B., Vongkaluang C., Sornnuwat Y., Lee C.-Y., Yoshimura T., Tsunoda K. (2012). Resistance of polyamide and polyethylene cable sheathings
to termites in Australia, Thailand, USA, Malaysia and Japan: A comparison
of four field assessment methods. International
Biodeterioration & Biodegradation.

[181] Yang S. S., Wu W. M., Bertocchini F., Benbow M. E., Devipriya S. P., Cha H. J., Peng B. Y., Ding M. Q., He L., Li M.-X (2024). Radical
innovation breakthroughs of biodegradation of plastics by insects:
history, present and future perspectives. Front.
Environ. Sci. Eng..

[182] O’Sullivan C., Clarke W., Lockington D. (2005). Sources of
Hydrogen Sulfide in Groundwater on Reclaimed Land. J. Environ. Eng..

[183] Bauske B., Goetz D. (1993). Effects of Deicing-Salts
on Heavy
Metal Mobility. Acta hydrochimica et hydrobiologica.

[184] Fay L., Shi X. (2012). Environmental Impacts of Chemicals for Snow and Ice
Control: State of the Knowledge. Water, Air,
& Soil Pollution.

[185] Zinder, S. Physiological ecology of methanogens. In Methanogenesis: Ecology, Physiology, Biochemistry and Genetics; Chapman and Hall, 1993.

[186] Kleinheinz G., Hernandez J. (2016). Comparison of two laboratory methods
for the determination of biomethane potential of organic feedstocks. J. Microbiol. Methods.

[187] Lankiewicz T. S., Choudhary H., Gao Y., Amer B., Lillington S. P., Leggieri P. A., Brown J. L., Swift C. L., Lipzen A., Na H. (2023). Lignin
deconstruction
by anaerobic fungi. Nature Microbiology.

[188] Wang X., Padgett J. M., De la Cruz F. B., Barlaz M. A. (2011). Wood Biodegradation
in Laboratory-Scale Landfills. Environ. Sci.
Technol..

[189] Ximenes F. A., Gardner W. D., Cowie A. L. (2008). The decomposition
of wood products in landfills in Sydney, Australia. Waste Management.

[190] Eleazer W. E., Odle W. S., Wang Y., Barlaz M. A. (1997). Biodegradability
of Municipal Solid Waste Components in Laboratory-Scale Landfills. Environ. Sci. Technol..

[191] Wang X., Barlaz M. A. (2016). Decomposition and
carbon storage
of hardwood and softwood branches in laboratory-scale landfills. Science of The Total Environment.

[192] Ximenes F., Björdal C., Cowie A., Barlaz M. (2015). The decay
of wood in landfills in contrasting climates in Australia. Waste Management.

[193] Towprayoon, S. ; Ishigaki, T. ; Chiemchaisri, C. ; Abdel-Aziz, A. O. Chapter 3: Soild Waste Disposal. In 2019 Refinement to the 2006 IPCC Guidelines for National Greenhouse Gas Inventories, 2019.

[194] Lohani, S. P. ; Havukainen, J. Anaerobic Digestion: Factors Affecting Anaerobic Digestion Process. In Waste Bioremediation; Varjani, S. J. , Gnansounou, E. , Gurunathan, B. , Pant, D. , Zakaria, Z. A. , Eds.; Springer Singapore, 2019; pp 343–359. 10.1007/978-981-10-7413-4_18.

[195] Merlin Christy P., Gopinath L. R., Divya D. (2014). A review on anaerobic
decomposition and enhancement of biogas production through enzymes
and microorganisms. Renewable and Sustainable
Energy Reviews.

[196] Chanton J., Abichou T., Langford C., Spokas K., Hater G., Green R., Goldsmith D., Barlaz M. A. (2011). Observations on the methane oxidation capacity of landfill
soils. Waste Management.

[197] Ginn T. R., Wood B. D., Nelson K. E., Scheibe T. D., Murphy E. M., Clement T. P. (2002). Processes in microbial
transport
in the natural subsurface. Advances in Water
Resources.

[198] Griffin, D. M. Water and Microbial Stress. In Advances in Microbial Ecology; Alexander, M. , Ed.; Springer US, 1981; pp 91–136. 10.1007/978-1-4615-8306-6_3.

[199] Albright W., Benson C., Waugh W. (2010). Water Balance Covers
for Waste
Containment: Principles and Practice.

[200] Hanson R. S., Hanson T. E. (1996). Methanotrophic bacteria. Microbiol Rev..

[201] Héry M., Singer A. C., Kumaresan D., Bodrossy L., Stralis-Pavese N., Prosser J. I., Thompson I. P., Murrell J. C. (2008). Effect of earthworms on the community structure of
active methanotrophic bacteria in a landfill cover soil. ISME Journal.

[202] Spokas K., Bogner J., Chanton J. P., Morcet M., Aran C., Graff C., Golvan Y. M., Hebe I. (2006). Methane mass
balance at three landfill sites: what is the efficiency of capture
by gas collection systems?. Waste Manag.

[203] Shukla P. N., Pandey K. D., Mishra V. K. (2013). Environmental Determinants
of Soil Methane Oxidation and Methanotrophs. Critical Reviews in Environmental Science and Technology.

[204] Visvanathan C., Pokhrel D., Cheimchaisri W., Hettiaratchi J. P. A., Wu J. S. (1999). Methanotrophic activities in tropical
landfill cover soils: effects of temperature, moisture content and
methane concentration. Waste Management &
Research.

[205] Apiwantragoon, P. Field Hydrologic Evaluation of Final Covers for Waste Containment. PhD Thesis, University of Wisconsin, Madison, WI, 2007.

[206] Gebert, J. ; Huber-Humer, M. ; Cabral, A. R. Design of Microbial Methane Oxidation Systems for Landfills [Review]. Frontiers in Environmental Science, 2022. 10. 10.3389/fenvs.2022.907562.

[207] Daniel, D. E. Earthen liners for land disposal facilities. Proceeding Paper, Part of: Geotechnical practice for waste disposal, 1987.

[208] Cornelius M. L., Osbrink W. L. (2011). Influence of dry
soil on the ability
of Formosan subterranean termites, Coptotermes formosanus, to locate
food sources. J. Insect Sci..

[209] Reinhard J., Kaib M. (2001). Trail Communication During Foraging
and Recruitment in the Subterranean Termite Reticulitermes santonensis
De Feytaud (Isoptera, Rhinotermitidae). Journal
of Insect Behavior.

[210] Koerner, R. M. Designing with Geosynthetics (6 ed., Vol. 1); Xlibris Publishing Co., 2012.

[211] Alpern B., Lemos de Sousa M. J. (2002). Documented international enquiry
on solid sedimentary fossil fuels; coal: definitions, classifications,
reserves-resources, and energy potential. International
Journal of Coal Geology.

[212] ISO . Classification of coals. In (Vol. ISO 11760), 2018.

[213] UNECE . International Classification of in-Seam Coals. 1998. https://digitallibrary.un.org/record/260911?ln=en&v=pdf.

[214] Barlaz M. A. (2006). Forest products decomposition in
municipal solid waste
landfills. Waste Management.

[215] Bogner, J. ; Vogt, M. ; Piorkowski, R. Landfill gas generation and migration: Review of current research II. Report Number # CONF-8901100–2. Conference: Solar Energy Research Institute (SERI) anaerobic digestion review meeting, Golden, CO, USA, 25 Jan 1989. https://www.osti.gov/servlets/purl/6347445.

[216] Gavrila G. O., Vasile G. G., Calinescu S. M., Constantin C., Tanase G., Cirstea A., Stancu V., Danciulescu V., Orbeci C. (2025). Assessment of CH_4_ and
CO_2_ Emissions from a Municipal Waste Landfill: Trends,
Dispersion, and Environmental Implications. Atmosphere.

[217] Wang J. M., Murphy J. G., Geddes J. A., Winsborough C. L., Basiliko N., Thomas S. C. (2013). Methane fluxes measured by eddy covariance
and static chamber techniques at a temperate forest in central Ontario,
Canada. Biogeosciences.

[218] Kholy S. M., Almetwally A. G., Mohamed I. M., Loloi M., Abou-Sayed A., Abou-Sayed O. (2018). A New Technique to Predict In-Situ
Stress Increment due to Slurry Injection into Sandstone Formations:
Case Study from a Biosolids Injector in Los Angeles, California, USA. SPE Western Regional Meeting.

[219] Panchal Y., Mounir N., Loloi M., Mohamed I., Abou-Sayed O., Abou-Sayed A. (2021). Application
of Slurry Injection Technology
in Biowaste Management - A New Discipline in Managing Bio-Waste in
Economic and Environmentally Friendly Manner. SPE Western Regional Meeting.

[220] Vaulted deep. Repurposing proven oil & gas technology. Retrieved June 20, 2025 from https://vaulteddeep.com/technology/.

[221] Pang B., Pang X., Li C., Chen Z., Xiao H., Hu S., Zhang S., Wang L., Sun Y., Li M., Hui S. (2024). A novel method
for quantitatively
identifying driving forces and evaluating their contributions to oil
and gas accumulation. Geoscience Frontiers.

[222] Schowalter T. T. (1979). Mechanics of Secondary Hydrocarbon
Migration and Entrapment. AAPG Bulletin.

[223] Bardalai M., Bordoloi N. K., Mahanta D. K. (2017). Production and biodegradability
analysis of bael shell pyrolysis oil. Materials
Today: Proceedings.

[224] Basaglia M., Favaro L., Torri C., Casella S. (2021). Is pyrolysis
bio-oil prone to microbial conversion into added-value products?. Renewable Energy.

[225] Reiners, P. ; Hiett, CD ; Nicholas, M. ; Chorover, J. ; Root, R. ; Guzman, A. . Geochemical and Mineralogical Reactions Between Bio-Oil Rocks, and Saline/brackish Groundwater: Implications for Subsurface Carbon Storage and Critical Metal Extraction. Geological Society of America Abstracts with Programs 2024. 56 (5). 10.1130/abs/2024AM-402351.

[226] Chukwuneke J. L., Ewulonu M. C., Chukwujike I. C., Okolie P. C. (2019). Physico-chemical analysis of pyrolyzed bio-oil from
swietenia macrophylla (mahogany) wood. Heliyon.

[227] Maulinda L., Husin H., Rahman N. A., Rosnelly C. M., Nasution F., Abidin N. Z., Faisal, Yani F. T., Ahmadi (2023). Effects of temperature and times
on the product distribution of bio-oils derived from Typha latifolia
pyrolysis as renewable energy. Results in Engineering.

[228] Jeffords, R. Graphic Representation of Oil-field Brines in Kansas. Kansas Geological Survey, 2024. 76. https://www.kgs.ku.edu/Publications/Bulletins/76_1/index.html. 10.17161/kgsbulletin.no.76.22000.

[229] Jo H., Verma D., Kim J. (2018). Excellent
aging stability of upgraded
fast pyrolysis bio-oil in supercritical ethanol. Fuel.

[230] Sun Z., Wang H., Zeng Y., Liu J., Maeda N. (2024). Understanding
and enhancing the phase stability of fast pyrolysis oils through ternary
phase diagrams. Chemical Engineering Journal.

[231] Black S., Ferrell J. R. (2020). Accelerated
aging of fast pyrolysis bio-oil: a new method based on carbonyl titration. RSC Adv..

[232] Mullen C. A., Boateng A. A. (2011). Characterization
of water insoluble
solids isolated from various biomass fast pyrolysis oils. Journal of Analytical and Applied Pyrolysis.

[233] Arnold S., Moss K., Henkel M., Hausmann R. (2017). Biotechnological
Perspectives of Pyrolysis Oil for a Bio-Based Economy. Trends Biotechnol..

[234] Dörsam, S. ; Kirchhoff, J. ; Bigalke, M. ; Dahmen, N. ; Syldatk, C. ; Ochsenreither, K. Evaluation of Pyrolysis Oil as Carbon Source for Fungal Fermentation. Frontiers in Microbiology 2016, 7. 10.3389/fmicb.2016.02059. PMC5177650 28066378

[235] Jarboe L. R., Wen Z., Choi D., Brown R. C. (2011). Hybrid
thermochemical processing: fermentation of pyrolysis-derived bio-oil. Appl. Microbiol. Biotechnol..

[236] Neumann, A. ; Dörsam, S. ; Oswald, F. ; Ochsenreither, K. Microbial Production of Value-Added Chemicals from Pyrolysis Oil and Syngas. In Sustainable Production of Bulk Chemicals: Integration of Bio-Chemo- Resources and Processes; Xian, M. , Ed.; Springer Netherlands, 2015; pp 69–105. 10.1007/978-94-017-7475-8_4.

[237] Parkes R. J., Wellsbury P., Mather I. D., Cobb S. J., Cragg B. A., Hornibrook E. R. C., Horsfield B. (2007). Temperature
activation of organic matter and minerals during burial has the potential
to sustain the deep biosphere over geological timescales. Org. Geochem..

[238] Jampolski L., Tomasi Morgano M., Seifert H., Kolb T., Willenbacher N. (2017). Flow Behavior
and Aging of Pyrolysis Oils from Different
Feedstocks. Energy Fuels.

[239] Oasmaa A., Fonts I., Pelaez-Samaniego M. R., Garcia-Perez M. E., Garcia-Perez M. (2016). Pyrolysis Oil Multiphase Behavior
and Phase Stability: A Review. Energy Fuels.

[240] Peralta, J. ; Raouf, M. A. ; Tang, S. ; Williams, R. C. Bio-Renewable Asphalt Modifiers and Asphalt Substitutes. In Sustainable Bioenergy and Bioproducts: Value Added Engineering Applications, Eds.; Springer London, 2012; pp 89–115. 10.1007/978-1-4471-2324-8_6.

[241] Foston M., Ragauskas A. J. (2012). Biomass
Characterization: Recent
Progress in Understanding Biomass Recalcitrance. Industrial Biotechnology.

[242] Himmel, M. A. , WS; Atalla, R. ; Bar-Peled, M. ; Bayer, E. ; Berry, A. ; Biomass Recalcitrance: Deconstructing the Plant Cell Wall for Bioenergy; Himmel, M. , Ed.; Wiley-Blackwell, 2009. 10.1002/9781444305418.

[243] McCann M. C., Carpita N. C. (2015). Biomass recalcitrance: a multi-scale,
multi-factor, and conversion-specific property. Journal of Experimental Botany.

[244] NREL . Biomass Compositional Analysis Laboratory Procedures. 2025. https://www.nrel.gov/bioenergy/biomass-compositional-analysis.

[245] NREL . Determination of Structural Carbohydrates and Lignin in Biomass: Laboratory Analytical Procedure (LAP); Sluiter, A. ; Hames, B. ; Ruiz, R. ; Scarlata, C. ; Sluiter, J. ; Templeton, D. ; Crocker, D. , Eds.; NREL, 2012.

[246] Fukushima R. S., Kerley M. S., Ramos M. H., Kallenbach R. L. (2021). The acetyl
bromide lignin method accurately quantitates lignin in forage. Animal Feed Science and Technology.

[247] del Río J. C., Rencoret J., Prinsen P., Martínez Á. T., Ralph J., Gutiérrez A. (2012). Structural
Characterization of Wheat
Straw Lignin as Revealed by Analytical Pyrolysis, 2D-NMR, and Reductive
Cleavage Methods. J. Agric. Food Chem..

[248] Happs R.
M., Addison B., Doeppke C., Donohoe B. S., Davis M. F., Harman-Ware A. E. (2021). Comparison
of methodologies used
to determine aromatic lignin unit ratios in lignocellulosic biomass. Biotechnol Biofuels.

[249] Addison B., Dickwella Widange M.
C., Pu Y., Ragauskas A. J., Harman-Ware A. E. (2025). Solid-state NMR at natural isotopic
abundance for bioenergy applications. Biotechnology
for Biofuels and Bioproducts.

[250] Ralph S. A., Ralph J., Lu F. (2024). NMR Database of Lignin and
Cell Wall Model Compounds.

[251] Pettersen, R. C. The Chemical Composition of Wood. In The Chemistry of Solid Wood; American Chemical Society, 1984; Vol. 207, pp 57–126. 10.1021/ba-1984-0207.ch002.

[252] Carpita, N. C. S. E. M. Linkage Structure of Carbohydrates by Gas Chromatography-Mass Spectrometry (GC-MS) of Partially Methylated Alditol Acetates. In Analysis of Carbohydrates by GLC and MS; Christopher, G. D. M. , Biermann, J. , Ed.; CRC Press, 1988, pp 60). CRC Press, 1988.

[253] Park S., Baker J. O., Himmel M. E., Parilla P. A., Johnson D. K. (2010). Cellulose crystallinity index: measurement
techniques
and their impact on interpreting cellulase performance. Biotechnology for Biofuels.

[254] Almendros G., Dorado J., González-Vila F. J., Blanco M. J., Lankes U. (2000). 13C NMR assessment of decomposition
patterns during composting of forest and shrub biomass. Soil Biology and Biochemistry.

[255] Bonanomi G., Incerti G., Giannino F., Mingo A., Lanzotti V., Mazzoleni S. (2013). Litter quality
assessed by solid
state 13C NMR spectroscopy predicts decay rate better than C/N and
Lignin/N ratios. Soil Biology and Biochemistry.

[256] Guerra F. P., Richards J. H., Fiehn O., Famula R., Stanton B. J., Shuren R., Sykes R., Davis M. F., Neale D. B. (2016). Analysis of the genetic variation
in growth, ecophysiology,
and chemical and metabolomic composition of wood of Populus trichocarpa
provenances. Tree Genetics & Genomes.

[257] Harman-Ware A. E., Orton K., Deng C., Kenrick S., Carpenter D., Ferrell J. R. (2020). Molecular weight
distribution of
raw and catalytic fast pyrolysis oils: comparison of analytical methodologies. RSC Adv..

[258] Sluiter, A. R. R. ; Scarlata, C. ; Sluiter, J. ; Templeton, D. Determination of Extractives in Biomass: Laboratory Analytical Procedure (LAP) 2008. https://docs.nrel.gov/docs/gen/fy08/42619.pdf.

[259] Khan S. R., Sharma B., Chawla P. A., Bhatia R. (2022). Inductively
Coupled Plasma Optical Emission Spectrometry (ICP-OES): a Powerful
Analytical Technique for Elemental Analysis. Food Analytical Methods.

[260] Martin M., Brice D., Martin S., André N., Labbé N. (2021). Inorganic characterization of switchgrass
biomass using
laser-induced breakdown spectroscopy. Spectrochimica
Acta Part B: Atomic Spectroscopy.

[261] Batstone D. J., Tait S., Starrenburg D. (2009). Estimation
of hydrolysis parameters in full-scale anerobic digesters. Biotechnol. Bioeng..

[262] Filer J., Ding H. H., Chang S. (2019). Biochemical
Methane
Potential (BMP) Assay Method for Anaerobic Digestion Research. Water.

[263] Amin F. R., Huang Y., He Y., Zhang R., Liu G., Chen C. (2016). Biochar applications
and modern techniques for characterization. Clean Technologies and Environmental Policy.

[264] ASTM International . Coal Standards and Gas Standards. Retrieved June 20, 2025 from https://store.astm.org/products-services/standards-and-publications/standards/coal-standards-and-gas-standards.html.

[265] Gleit, A. Coal sampling and analysis: methods and models, 1985. https://nepis.epa.gov/.

[266] ISO . Coals: Including Lignites. Retrieved June 20, 2025 from https://www.iso.org/ics/73.040/x/.

[267] Zhu, Q. Coal sampling and analysis standards, 2014. https://usea.org/sites/default/files/042014_Coal%20sampling%20and%20analysis%20standards_ccc235.pdf.

[268] Synnott D. P., Sanei H., Dewing K., Ardakani O. H., Pedersen P. K. (2017). Insight into visible light spectrum
changes with increasing
reflectance in bituminite and inertinite macerals. Fuel.

[269] European Biochar Certification . Analytical Methods. European Biochar Certificate (EBC), 2024. https://www.european-biochar.org/en/ct/8-Analytical-Methods.

[270] Brewer C. E., Schmidt-Rohr K., Satrio J. A., Brown R. C. (2009). Characterization
of biochar from fast pyrolysis and gasification systems. Environmental Progress & Sustainable Energy.

[271] Sigmund G., Hüffer T., Hofmann T., Kah M. (2017). Biochar total
surface area and total pore volume determined by N2 and CO2 physisorption
are strongly influenced by degassing temperature. Science of The Total Environment.

[272] Yargicoglu E. N., Sadasivam B. Y., Reddy K. R., Spokas K. (2015). Physical and
chemical characterization of waste wood derived biochars. Waste Management.

[273] Brewer C. E., Chuang V. J., Masiello C. A., Gonnermann H., Gao X., Dugan B., Driver L. E., Panzacchi P., Zygourakis K., Davies C. A. (2014). New approaches to measuring biochar
density and porosity. Biomass and Bioenergy.

[274] Khuong, D. A. ; Nguyen, H. N. Engineered Biochar as Gas Adsorbent. In Engineered Biochar: Fundamentals, Preparation, Characterization and Applications; Ramola, S. , Mohan, D. , Masek, O. , Méndez, A. , Tsubota, T. , Eds.; Springer Nature Singapore, 2022; pp 237–258). 10.1007/978-981-19-2488-0_13.

[275] Wen C., Liu T., Wang D., Wang Y., Chen H., Luo G., Zhou Z., Li C., Xu M. (2023). Biochar as the effective
adsorbent to combustion gaseous pollutants: Preparation, activation,
functionalization and the adsorption mechanisms. Prog. Energy Combust. Sci..

[276] Kanaujia P. K., Sharma Y. K., Agrawal U. C., Garg M. O. (2013). Analytical
approaches to characterizing pyrolysis oil from biomass. Trends Anal. Chem..

[277] Oasmaa, A. ; Leppaemaeki, E. ; Koponen, P. ; Levander, J. ; Tapola, E. Physical characterization of biomass-based pyrolysis liquids. Application of standard fuel oil analyses. 1997. https://www.osti.gov/etdeweb/servlets/purl/594319.

[278] Sipilä K., Kuoppala E., Fagernäs L., Oasmaa A. (1998). Characterization of biomass-based flash pyrolysis oils. Biomass and Bioenergy.

[279] Miscall, J. ; Christensen, E. D. ; Olstad, J. ; Deutch, S. ; Ferrell, J. R., III Determination of Carbon, Hydrogen, Nitrogen, and Oxygen in Bio-Oils: Laboratory Analytical Procedure (LAP) Technical Report NREL/TP-5100-80967, 2021. https://www.nrel.gov/docs/fy22osti/80967.pdf.

[280] Nolte M. W., Liberatore M. W. (2010). Viscosity
of Biomass Pyrolysis Oils
from Various Feedstocks. Energy Fuels.

[281] Black, S. ; Christensen, E. ; Ferrell, J. R., III . Accelerated Aging of Fast Pyrolysis Bio-Oil Using Carbonyl Titration: Laboratory Analytical Procedure (LAP), 2021. https://www.nrel.gov/docs/fy22osti/80966.pdf.

[282] Happs, R. M. ; Harman-Ware, A. E. ; Ben, H. ; Ferrell, J. R., III Determination of Carbon Functional Groups in Pyrolysis Bio-Oils using 13C NMR: Laboratory Analytical Procedure (LAP); National Renewable Energy Laboratory: Golden, CO, 2021.

[283] Olarte, M. B. ; SD; Swita, M. ; Padmaperuma, A. B. ; Ferrell, J. ; Ben, H. Determination of Hydroxyl Groups in Pyrolysis Bio-oils using 31P NMR: Laboratory Analytical Procedure (LAP), 2016. https://docs.nrel.gov/docs/fy16osti/65887.pdf.

[284] Christensen, E. ; Ferrell, J. ; Olarte, M. V. ; Padmaperuma, A. B. Quantification of Semi-Volatile Oxygenated Components of Pyrolysis Bio-Oil by Gas Chromatography/Mass Spectrometry (GC/MS): Laboratory Analytical Procedure (LAP), 2016. https://docs.nrel.gov/docs/fy16osti/65889.pdf.

[285] Black B. A. (2016). Aqueous Stream Characterization
from Biomass
Fast Pyrolysis and Catalytic Fast Pyrolysis. ACS Sustainable Chem. Eng..

[286] Harman-Ware A. E., Ferrell J. R. (2018). Methods and Challenges
in the Determination of Molecular Weight Metrics of Bio-oils. Energy Fuels.

[287] Mullen C. A., Strahan G. D., Boateng A. A. (2019). Characterization
of Biomass Pyrolysis Oils by Diffusion Ordered NMR Spectroscopy. ACS Sustainable Chem. Eng..

[288] Sparrow K. J. (2019). Stable isotopic determination
of methane oxidation:
When smaller scales are better. Waste Management.

[289] Rutqvist J. (2012). The Geomechanics of CO2 Storage in
Deep Sedimentary
Formations. Geotechnical and Geological Engineering.

[290] Valluri M., Mishra S., Ravi Ganesh P. (2021). Injectivity
index: a powerful tool for characterizing CO2 storage reservoirs—a
technical note. Greenhouse Gases: Science and
Technology.

[291] Isometric . Biomass Geological Storage. BiCRS Protocol 2024 April 1, 2025].

[292] Prensky, S. E. Advances in borehole imaging technology and applications. In Borehole Imaging: applications and case histories; Lovell, M. A. , Williamson, G. , Harvey, P. K. , Eds.; Geological Society of London Special Publications: London, England, v. 159, pp 1–43, 1999. 10.1144/GSL.SP.1999.159.01.01.

[293] Smith N. T., Shreeve J., Kuras O. (2020). Multi-sensor
core logging
(MSCL) and X-ray computed tomography imaging of borehole core to aid
3D geological modelling of poorly exposed unconsolidated superficial
sediments underlying complex industrial sites: An example from Sellafield
nuclear site, UK. Journal of Applied Geophysics.

[294] Conaway C. H., Thordsen J. J., Manning M. A., Cook P. J., Trautz R. C., Thomas B., Kharaka Y. K. (2016). Comparison
of geochemical
data obtained using four brine sampling methods at the SECARB Phase
III Anthropogenic Test CO2 injection site, Citronelle Oil Field, Alabama. International Journal of Coal Geology.

[295] Goodwin D. G., Shen S.-J., Lyu Y., Lankone R., Barrios A. C., Kabir S., Perreault F., Wohlleben W., Nguyen T., Sung L. (2020). Graphene/polymer nanocomposite
degradation by ultraviolet light: The effects of graphene nanofillers
and their potential for release. Polym. Degrad.
Stab..

